# Intrinsic limits to gene regulation by global crosstalk

**DOI:** 10.1038/ncomms12307

**Published:** 2016-08-04

**Authors:** Tamar Friedlander, Roshan Prizak, Călin C. Guet, Nicholas H. Barton, Gašper Tkačik

**Affiliations:** 1 Institute of Science and Technology Austria, Am Campus 1, A-3400 Klosterneuburg, Austria

## Abstract

Gene regulation relies on the specificity of transcription factor (TF)–DNA interactions. Limited specificity may lead to crosstalk: a regulatory state in which a gene is either incorrectly activated due to noncognate TF–DNA interactions or remains erroneously inactive. As each TF can have numerous interactions with noncognate *cis*-regulatory elements, crosstalk is inherently a global problem, yet has previously not been studied as such. We construct a theoretical framework to analyse the effects of global crosstalk on gene regulation. We find that crosstalk presents a significant challenge for organisms with low-specificity TFs, such as metazoans. Crosstalk is not easily mitigated by known regulatory schemes acting at equilibrium, including variants of cooperativity and combinatorial regulation. Our results suggest that crosstalk imposes a previously unexplored global constraint on the functioning and evolution of regulatory networks, which is qualitatively distinct from the known constraints that act at the level of individual gene regulatory elements.

## Abstract (web summary)

Limited specificity of transcription factor-DNA interactions leads to crosstalk in gene regulation. Here the authors consider global crosstalk in regulatory networks of growing size and complexity, and show that it imposes constraints on gene regulation and on the evolution of regulatory networks.

## Graphical abstract

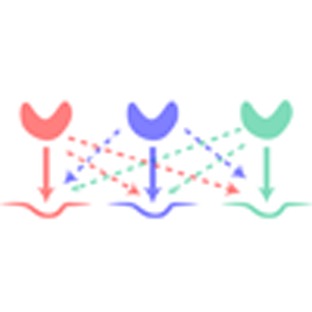

Life depends on the specificity of molecular recognition to ensure that essential reactions only occur between cognate substrates even when similar non-cognate substrates are present, sometimes in large excess. A paradigmatic example is that of the aminoacyl transfer RNA synthetase^1^, which uses kinetic proofreading^2^ to load appropriate amino acids onto matching tRNAs. This and other examples—including DNA replication, ligand sensing^3^, protein–protein interactions^4^,^5^,^6^,^7^,^8^,^9^, recognition events in the immune system^10^,^11^ and molecular self-assembly^12^—indicate that biology places a large premium on the reduction of unintended ‘crosstalk', a generic term that encompasses all potentially disruptive processes due to reactions between non-cognate substrates.

Molecular recognition is fundamental also to transcriptional regulation, the primary mechanism by which cells control gene expression. The specificity of this regulation ultimately originates in the binding interactions between special regulatory proteins, called transcription factors (TFs), and short regulatory sequences on the DNA, called binding sites. Although each type of TF preferentially binds certain regulatory DNA sequences, a large body of evidence shows that this binding specificity is limited, and that TFs bind other non-cognate targets as well^13^,^14^,^15^,^16^,^17^. These additional binding targets were previously discussed in the context of their effect on the TF concentration^18^,^19^. However, if these sites happen to also be regulatory elements of other genes, non-cognate binding not only depletes TF molecules but could also actively interfere with gene regulation. This suggests that the crosstalk problem is global: in a pool of TF molecules of different chemical species co-expressed at any one time, each molecule has a small probability of erroneously regulating some subset of all genes. As the regulatory system grows in complexity, the number of potential non-cognate interactions will grow faster than the number of cognate interactions. Although this makes the problem biologically relevant and theoretically interesting, existing work has mostly considered a reduced setting, by computing binding probabilities for a single TF to cognate versus non-cognate sites^20^,^21^,^22^,^23^. Such a reduced description thus overlooked the effect of this TF on the (mis)regulation of genes that were not its cognate regulatory targets. Motivated by this observation, our primary goal here is to develop a new framework for crosstalk that captures its global nature, by simultaneously treating multiple TFs and multiple regulatory binding sites. Moreover, the focus of prior work has been on how to achieve reliable gene regulation by cognate TFs^24^, whereas the complementary question of how to prevent erroneous regulation by non-cognate TFs has remained largely unexplored (but see ref. 25). As a result, it remains unclear whether crosstalk places strong constraints on the ability of cells to orchestrate their gene expression programmes, and to what extent different molecular mechanisms could relax any such constraints.

A model of crosstalk in transcriptional regulation should satisfy three key requirements for biophysical plausibility. First, the model should be global. Global models, where many targets are simultaneously regulated by different TFs, will properly capture the faster-than-linear growth in the number of possible non-cognate interactions as the number of TFs increases, and the difficulty in ensuring that recognition sequences for all TFs remain sufficiently distinct. Second, the model should explicitly account for differential activation of genes depending on regulatory conditions. Consequently—and in contrast to previously studied cases of molecular recognition^2^—the distinction between ‘erroneous' and ‘correct' outcomes of regulation will depend on the presence/absence of the regulatory signals. In particular, the ability of the regulatory system to keep genes reliably inactive when appropriate, despite crosstalk interference, will emerge as an important consideration. Third, textbook models of transcriptional regulation assume that TF–DNA interactions happen in equilibrium^22^,^26^. This assumption, which is supported experimentally for prokaryotic regulation^27^,^28^ and which underlies the majority of modelling and bioinformatic applications, puts strong constraints on models of crosstalk. In this work, we explore its consequences in depth; we report on out-of-equilibrium schemes elsewhere^29^.

Here we construct a biophysical model for crosstalk in transcriptional regulation that accounts for all cross-interactions between regulators and their binding sites. We identify the parameters that have a major influence on crosstalk severity. Although some of these parameters, such as the free concentration of TFs, are difficult to estimate, we show that there exists a lower bound to crosstalk with respect to these parameters. This implies the existence of a ‘crosstalk floor', which cannot be overcome even if TF concentrations were optimally adjusted by the cell, by various feedback mechanisms or otherwise, and compensated for sequestration at non-cognate sites. Our model allows us to ask a number of fundamental questions: How does the severity of crosstalk depend on the number of (co-expressed) genes or the biophysical properties of TF–DNA interactions, such as binding site length and binding energy, for which we have reliable estimates? How do the regulatory strategies of prokaryotes compare with those of eukaryotes? Do complex regulatory schemes, such as combinatorial regulation by activators and repressors, or cooperative activation, lower crosstalk, as is often implied^24^? Many biophysical constraints have been shown to shape the properties of genetic regulatory networks, for example, programmability^20^, response speed^30^, noise in gene expression and dynamic range of regulation^31^,^32^,^33^,^34^, robustness^35^ and evolvability of the regulatory sequences^36^,^37^. Most of these constraints, however, could be understood at the level of individual genetic regulatory elements. We find that crosstalk is special: although it originates locally due to biophysical limits to molecular recognition, its cumulative effect only emerges globally. At the level of a single genetic regulatory element, crosstalk can always be avoided by increasing the concentration of cognate TFs or introducing multiple binding sites in the promoter. It is only when we self-consistently consider that these same cognate TFs act as non-cognate TFs for other genes, or that new binding sites in the promoter drastically increase the number of non-cognate binding configurations, that crosstalk constraints become clear.

## Results

### A thermodynamic model of global crosstalk

We start by introducing a basic model of regulation, in which each gene will be regulated in the simplest possible manner by a dedicated TF type and the mechanism of regulation will be identical for every gene. For this basic model, where the limits to crosstalk are analytically computable, we will outline the reasoning, sketch the derivation and interpret the results in the main text. We will then relax our simplifying assumptions in a variety of ways and extend the analysis to more elaborate regulatory schemes, such as different flavours of cooperative or combinatorial regulation. We will summarize the corresponding results later in this section and present detailed computations in the Supplementary Information.

**Figure 1:**
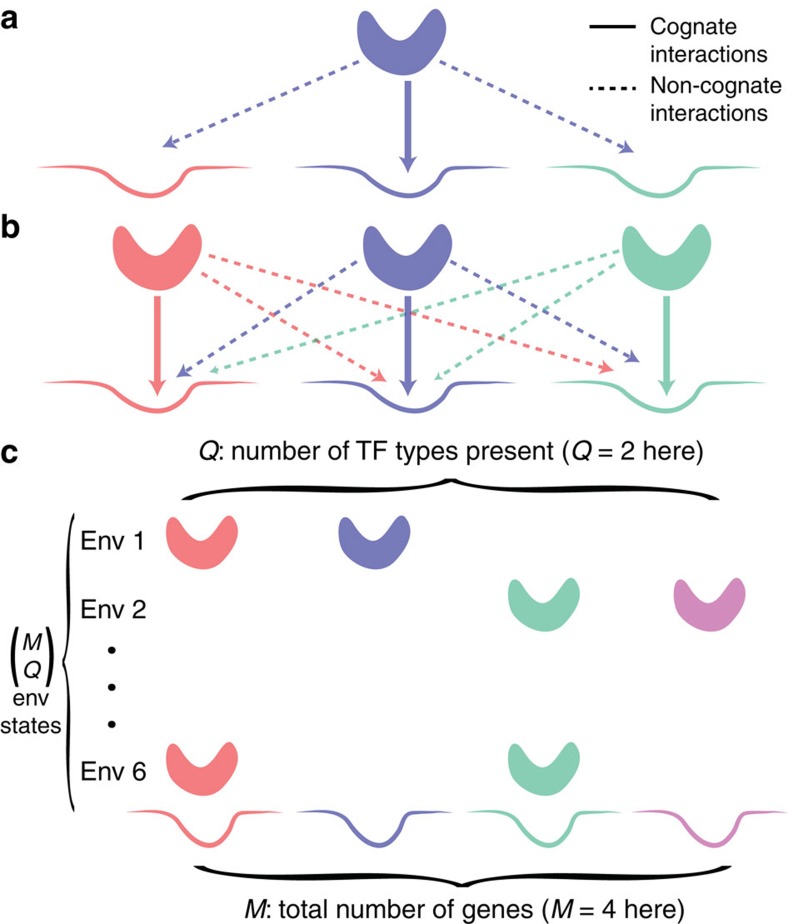
Crosstalk in gene regulation. (**a**) A TF preferentially binds to its cognate binding site, but can also bind non-cognate sites, potentially causing crosstalk—an erroneous activation or repression of a gene. (**b**) In a global setting where many TFs regulate many genes, the number of possible non-cognate interactions grows quickly with the number of TFs; in addition, it may become difficult to keep TF recognition sequences sufficiently distinct from each other. (**c**) Cells respond to changing environments by attempting to activate subsets of their genes. In this example, the total number of genes is *M*=4 and different environments (here, 6 in total) call for activation of different subsets with *Q*=2 genes. To control the expression in every environment, TFs for *Q* required genes are present, whereas the TFs for the remaining *M*−*Q* genes are absent. Because of crosstalk, TFs can bind non-cognate sites, generating a pattern of gene expression that can differ from the one required.

We consider a cell that contains *M* genes, which need to be transcriptionally regulated. In the basic model, each gene is associated with a single binding site of length *L* basepairs and a unique kind of TF, which—in environments where the TF is expressed—preferably binds to that binding site to activate transcription. We assume that the genes are inactive, unless a TF binds to their binding site. We later relax this simplification to cases where each TF regulates several genes. Every TF can also bind other (non-cognate) binding sites, albeit with lower probability, as schematized in Fig. 1a, b. These non-cognate interactions will contribute to crosstalk in our model.

Gene regulation gives cells the ability to differentially activate subsets of their genes in a manner appropriate to the environmental conditions, signals, cell type or time. In our basic model, we imagine a cell that responds to different environments by activating different subsets of *Q* genes (out of a total of *M* genes), while keeping the remaining *M*−*Q* genes inactive (see Fig. 1c). As regulation unfolds, the regulatory network thus switches between equilibrium states where any choice of *Q* out of *M* genes could be activated; to make the problem tractable, we assume that all these choices are equally probable. In a given environment, activating a particular subset of *Q* out of *M* genes is achieved by expressing the corresponding *Q* TF types. The remaining *M*−*Q* TF types, corresponding to the genes that should remain inactive, are absent in the cell.

How does the cell express the correct set of TFs for any particular environment and at what concentrations are these TFs expressed? The issue is made seemingly even more complicated by the fact that the TF concentration reflects the total number of TF molecules in the cell, as well as any possible effects due to nonspecific TF localization or sequestration on the DNA and elsewhere^18^,^19^,^38^. What we will show below is that even if the TF presence and concentrations were perfectly adjusted to the environment, a residual level of crosstalk—representing a lower bound or intrinsic limit—is inevitable. As we are interested precisely in this limit, we will not need to specify the mechanisms by which cells control their TF concentrations, which probably involve complex regulatory network dynamics with feedback loops; instead, we will mathematically look for the lowest achievable crosstalk and show that even in an optimal scenario crosstalk can present a serious regulatory problem.

Using the mismatch energy model to describe the interactions of TFs with their binding sites and basic statistical mechanics^20^,^39^, we can compute crosstalk, *X*, which we define to be the average fraction of all genes in erroneous regulatory states (see Methods). Erroneously regulated genes are genes that should be expressed in a given environment but are not, because their corresponding TFs failed to bind the required activator sites; genes that should be unexpressed in a given environment but are erroneously induced due to spurious binding of non-cognate TFs; or genes that should be expressed, but are expressed due to binding of non-cognate (instead of cognate) TFs. Global crosstalk *X* ranges between zero (no erroneous regulation) and one (every gene is mis-regulated), and depends on the total number of genes in an organism, *M*, the typical number of co-activated genes in every environment, *Q*, the concentration of TFs when they are present, *C*, and the binding specificity of the TFs for different regulatory sequences (Equations (3)–(5), 19, 20).

**Figure 2:**
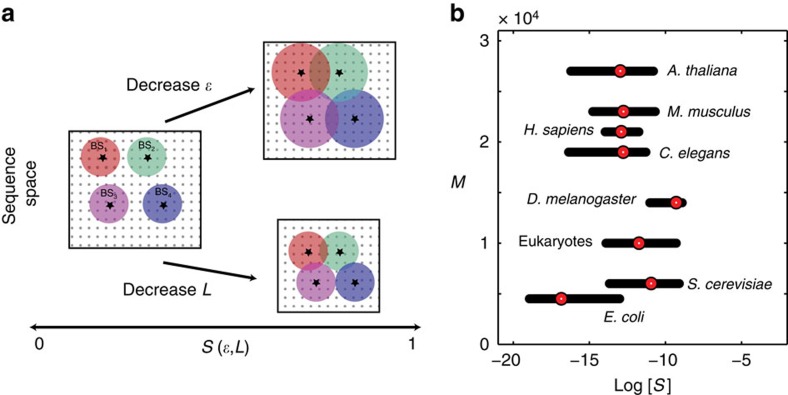
Binding site similarity *S* and number of genes *M* are basic determinants of crosstalk. (**a**) Binding site similarity, 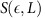, determines the likelihood that a TF will bind non-cognate sites, if recognition sequences are of length *L* and the energy per mismatch is 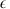. A schematic diagram of sequence space packing by different TFs: sequences (dots) in a coloured circle are likely to be bound by the TF whose consensus is the circle's centre star. Smaller *L* contracts the sequence space and makes crosstalk (circle overlap) more likely (larger *S*); crosstalk is increased (larger *S*) also by smaller 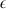, which expands the circle radius. (**b**) Typical values for the number of genes, *M*, and binding site similarity, 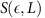, across different taxa, estimated from genomic databases. For each organism, we find a distribution of *S* over its reported TFs (dots=median of the distribution, black bars=±1-quartile range; see Supplementary Note 2 for details).

The major determinant of crosstalk is the likelihood of TFs to bind non-cognate sites, which is determined by the similarity between cognate and non-cognate sites. In the global setting, making a particular site less similar to all the remaining sites can only happen at the cost of making the remaining sites more similar among themselves. For a large number of sites we describe this effect by introducing an average binding site similarity measure *S*_*i*_ between the binding site of gene *i* and all others, defined as:


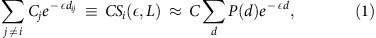


where *P*(*d*) is the distribution of mismatches between all pairs of binding sites in our model and *C* is the total concentration of all TFs. In the following we assume full symmetry between the genes, so that for every *i*, *S*_*i*_=*S*. *S* depends solely on the binding sites, but it carries no functional meaning in the absence of any TF, namely when *C*=0. We emphasize that this quantity, *S*, is not arbitrary, but rather emerges from our calculations in equations (3) and (4); a related measure of the likelihood of olfactory or immune receptors to bind an arbitrary ligand from a large repertoire has been previously introduced and measured^40^. *S*_*i*_ is proportional to the probability of the *i*-th TF to bind any non-cognate binding site. The highest level of similarity, *S*=1, occurs if all sites are identical (*d*=0). Similarity is very low, *S*≈0, if the sites are all significantly different from each other. The shorter the binding site length *L* is and the weaker the binding energy 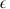, the larger *S* gets and the less distinguishable the sites are (Fig. 2a); simultaneously, we expect the crosstalk to increase, an intuition we will make precise in the following section.

Binding site similarity 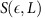 of equation (1) could be directly measured, by experimentally probing the average TF-binding affinity to a large repertoire of known binding sites. Alternatively, *S* can be estimated from bioinformatic data. In Fig. 2b we used databases of known TF-binding sites to extract organism-specific estimates for *S*. Under certain assumptions about how binding sites are organized in sequence space, *S* can be also computed theoretically. If the binding sites were random sequences of length *L*, one can derive a simple analytical expression for *S* (see Supplementary Note 1): 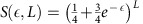. We also studied more realistic models for how TF-binding sites are organized, for example, taking into account the possibility of TFs to bind reverse-complemented sites, an improved biophysical model for mismatch energy that saturates with the number of mismatches and a model of binding sites that have evolved to be maximally distinct (Supplementary Note 2). All these variations ultimately only affect the value of *S*, while leaving the crosstalk formalism unchanged. We therefore carried out our main computations as a function of *S* directly. To estimate typical crosstalk for values of *S* that are biophysically realistic, we assumed that binding sites are distributed as randomly as possible in the sequence space, while avoiding excessive similarity (that is, we used the results of Supplementary Fig. 7 with *d*_min_=2).

### Basic crosstalk model exhibits three regulatory regimes

Although we can reasonably estimate the major determinants of crosstalk in our model—the number of genes typically coactivated, *Q*, the total number of genes, *M*, and the binding site similarity *S*—it is harder to determine the appropriate value for the total concentration of available TFs, *C*. This is not only due to the lack of quantitative data, but also because the relation between the total copy number of TFs in a cell and the concentration of TFs that are available for binding may be complicated^18^. We thus opted for an alternative approach: we look for a concentration C* that minimizes the crosstalk error *X*. An optimal C* emerges as a trade-off between activating the *Q* genes that should be active (for which a higher concentration is beneficial) and avoiding the activation of the remaining *M*−*Q* genes (for which a lower concentration is beneficial). Such a minimum, X*=*X*(C*), is a lower bound on crosstalk, which can be analytically computed in the mean field approximation (Supplementary Note 1), as well as validated numerically by simulation (Supplementary Note 3). This level of crosstalk cannot be decreased even if a cell could perfectly adjust its TF concentrations to the environment and optimally compensate the concentrations for nonspecific binding and sequestration.

**Figure 3:**
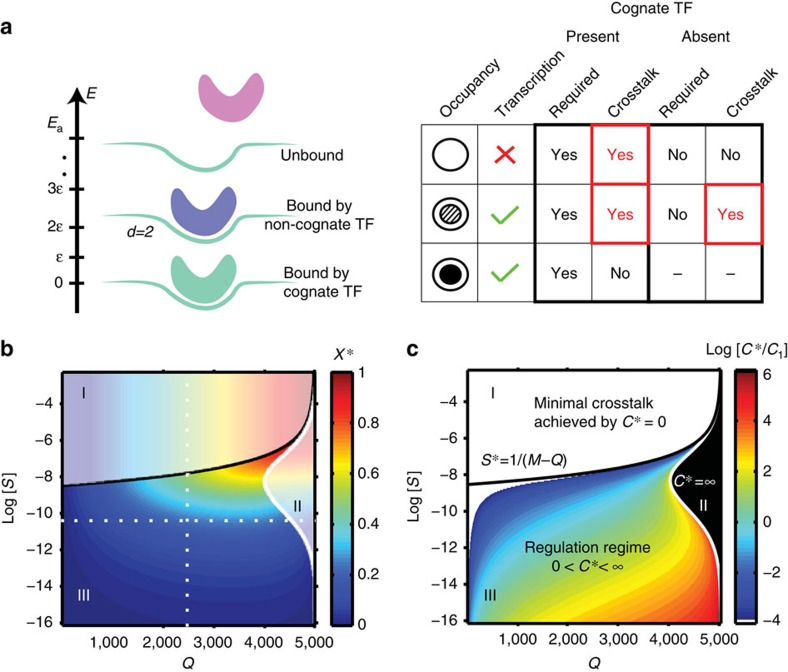
Basic model with one activator binding site per gene exhibits three distinct regulatory regimes. (**a**) Each binding site can be in either of the three possible states with different corresponding energies: bound by a cognate factor (*E*=0, green molecule), bound by a non-cognate factor with *d*-mismatches (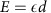, here a blue molecule with *d*=2), or unbound (*E*=*E*_*a*_, pink molecule). The table shows which of these states lead to transcription and which of these outcomes is considered as crosstalk when the cognate TF is present and the gene is required to be active (left), or if it is absent and the gene is required to be inactive (right). (**b**) Minimal crosstalk X*, shown in colour, as a function of the number of coactivated genes *Q* and binding site similarity, *S*. Three different regulatory regimes are separated by black and white boundary lines (analytical expressions in Supplementary Note 1), identical between **b** and **c**. Dotted lines refer to the ‘baseline parameters' (*Q*=2,500, *M*=5,000, log(*S*)=−10.5—represents *L*=10, 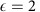 with *d*_min_=2) that we use in all subsequent figures if not specified differently. (**c**) Optimal TF concentration, C*, that minimizes the crosstalk, relative to *C*_1_, the optimal concentration at baseline parameters. For high binding site similarity (large *S*), the crosstalk is minimized at C*=0 (white region, I: ‘no regulation regime'). For *Q*→*M* and intermediate *S*, the crosstalk is minimized at C*→∞ (black region, II: ‘constitutive regime'). In a large, biologically plausible intermediate regime, crosstalk is minimized at a finite non-zero TF concentration (colour, III: ‘regulation regime').

First, we consider a fixed number of total genes, *M*=5,000, and ask how crosstalk depends on the number of co-activated genes, *Q*, and the binding site similarity, *S*, in our basic model, summarized in Fig. 3a. The optimization yields three distinct regulatory regimes, illustrated in Fig. 3b, c. For larger values of *S* where binding sites are very similar, regulation is so nonspecific that crosstalk is minimized by having no TF at all, that is, at C*=0 (region I). This regime, which happens whenever *S*>1/(*M*−*Q*), is dysfunctional and thus biologically implausible. Interestingly, the resulting fundamental limit to *S*, or to how similar binding sites can ever get while still permitting functional regulation, is set by *M*−*Q*, the typical number of genes that must remain inactive in each environment. This highlights the strong constraint on the regulatory system of keeping undesired gene activation levels low despite crosstalk interference.

As the organism tries to activate increasingly large subsets of genes in each environment and *Q* increases, the optimal concentration C* climbs until we reach a regime where C* formally diverges (region II), shown in Fig. 3c and Supplementary Fig. 2. In this limit, however, a biologically plausible solution would simply be to constitutively express the majority of the genes rather than using transcriptional regulation to do so, thus avoiding any possible crosstalk interference. This strategy might be applicable for organisms living in nearly constant environments, such as obligatory parasites.

Finally, there is a broad region (region III) in the (*S*, *Q*) plane where crosstalk is minimized by a finite positive value for the optimal TF concentration. In this regime, which we call the ‘regulation regime', as it corresponds to the biological notion of regulation, crosstalk is given to a very good approximation by


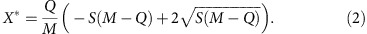


This simple expression for X* is one of our key results. It is independent of the energy gap between cognate and unbound states, *E*_a_; increasing this gap only lowers the optimal concentration, C*, while leaving the crosstalk unchanged. The crosstalk depends both on the fraction of genes that need to be activated, *Q*/*M*, as well as on the total number of genes that need to be inactive, *M*−*Q*, in a typical environment. This dependence also suggests that it is costly to maintain genes that are never expressed, arguing against unlimited accumulation of obsolete genes in organisms. Crosstalk X* in the regulation regime is dominated by the second term of equation (2) and thus increases as 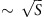 and as 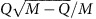 for sufficiently small *S*. At the boundary between regions I and III, where regulation breaks down, *S*(*M*−*Q*)=1; hence, X*=*Q*/*M* and is independent of *S* throughout region I, because all genes that need to be active are in a crosstalk state due to the absence of TFs. Alternatively, we can view equation (2) as a function of *M*, the total number of genes, at a fixed fraction of genes typically activated, *Q*/*M*. In that case we can see that the average binding site similarity, *S*, sets the limit to the maximum number of genes in the organism, *M*≲1/*S*, if the system is to stay in region III where regulation is effective. This is confirmed in Supplementary Fig. 1 by a detailed analysis of crosstalk for an organism with *M*=20,000 genes, a typical number for a metazoan.

A quick inspection of Fig. 3b shows that crosstalk in the basic model is surprisingly high for an organism of *M*=5,000 genes of which typically a half (*Q*=*M*/2) would be activated in each environment and with TF specificity typical of metazoans (log(*S*)=−10.5). At these ‘baseline' parameters, the crosstalk limit is X*≈0.23, implying that almost a quarter of the genes at any time would be in an erroneous regulatory state. This suggests that global crosstalk is a serious constraint, and that more complex regulatory mechanisms have evolved, in part, to permit reliable regulation despite non-cognate TF binding.

We also examined a number of variations of the basic model: (i) a variant where the expression of unnecessary proteins is not penalized equally to the incorrect expression of the necessary proteins (Supplementary Note 1); (ii) a variant where some genes are more ‘important' than others and thus should preferentially have small crosstalk errors (Supplementary Fig. 6); (iii) a variant where each TF can regulate Θ genes, which decreases the crosstalk by a factor of 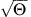 (Supplementary Note 1); and (iv) a variant where repression (Supplementary Note 1), or a mix of repression and activation (Supplementary Note 4), is used to regulate downstream genes. The results are detailed in the Supplementary Information and are summarized in Table 1.

### Crosstalk is not mitigated by complex regulatory schemes

So far, we considered the simplest *cis*-regulatory element architecture with a single TF binding site. Most genes, especially in eukaryotes, employ complex regulatory elements with multiple TF binding sites, some of which have been suggested in the literature to increase the effective binding specificity of TFs or protect the binding sites from spurious binding^24^,^25^. By implication, such effects are expected to also reduce crosstalk. We next use our theoretical framework to study quantitatively under what conditions that may be the case.

**Figure 4:**
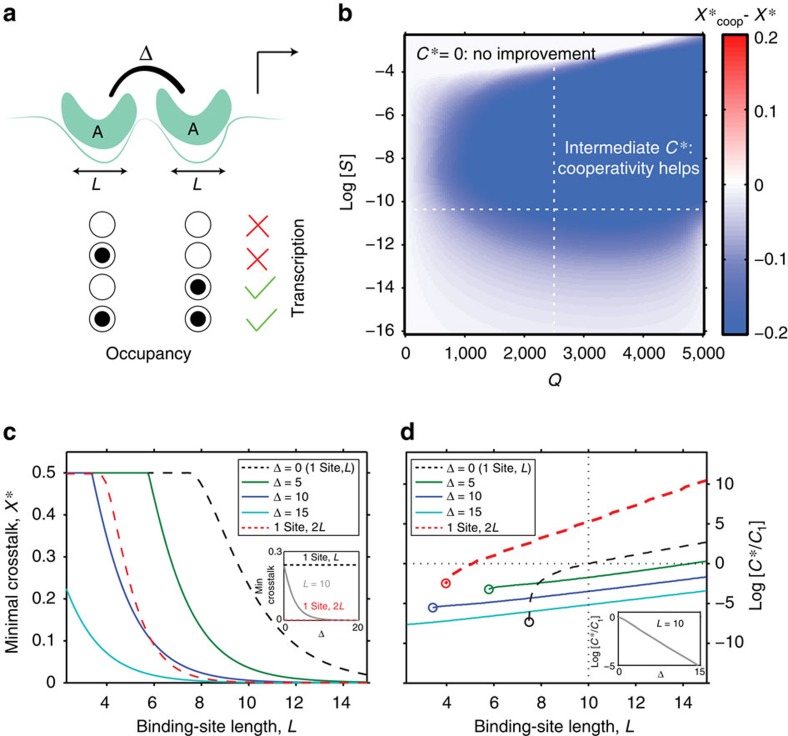
Cooperative regulation reduces crosstalk and the required optimal TF concentration. (**a**) Cognate binding configurations (non-cognate not shown) for two sites of length *L* leading to transcription (green check) or not (red cross); doubly occupied promoter gains a cooperative energy Δ. Transcription proceeds only when the proximal (rightmost) site is occupied. (**b**) Difference in minimal crosstalk, shown in colour, between the cooperative model and the basic model of Fig. 3, 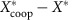, for cooperative interaction strength Δ=10. Cooperativity significantly reduces crosstalk (blue; at baseline parameters shown with white dashed lines, 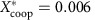 here versus X*=0.23 in the basic model) and shrinks the ‘no regulation' (C*=0) regime. (**c**) Minimal crosstalk error, X*, versus binding site length *L* for different values of cooperative energy Δ shows that strong cooperativity can decrease the crosstalk beyond the basic model with binding site of length 2*L* (red). (**d**) Optimal TF concentration, C*, required to minimize crosstalk, decreases with increasing cooperativity Δ for all *L* and is consistently below the single-site basic model with site length of either *L* (black) or even 2*L* (red). Circles denote transition to the ‘no regulation' (C*=0) regime at low *L* (large *S*), showing that cooperativity extends the ‘regulation regime.' In **c**,**d**, we convert *S* values to the equivalent binding site lengths *L* using the random sequence model.

We extend our basic model such that each gene is influenced by two nearby binding sites of length *L* to which cognate TFs can bind cooperatively. For simplicity, we assume that cooperativity occurs between TFs of the same type, although the framework can be extended to more general cases. This molecular configuration of two cognate DNA-bound proteins is favoured by an additional energy contribution Δ. We assume that only one of the two sites controls transcriptional activity directly (here, the site proximal to the gene start, for example, by polymerase recruitment^27^), whereas the other—here, the distal site—helps stabilize the binding to the proximal site, as schematized in Fig. 4a. In this model, as Δ goes to zero, the distal binding site has no effect on regulation and we recover the basic model of regulation by a single binding site (Fig. 3).

To assess whether cooperative regulation can reduce crosstalk, we compute the minimal achievable crosstalk, 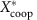, and compare this in Fig. 4b with the minimal crosstalk of the basic model, X*. We find that cooperativity can significantly reduce crosstalk in a large part of the ‘regulation regime,' which itself extends towards larger *S*. Examining in detail how the crosstalk behaves in Fig. 4c, we see that at a fixed binding site length *L*, minimization of crosstalk prefers strong cooperativity Δ; nevertheless, the improvement in crosstalk is bounded and as Δ grows, saturates at a limiting value. In this limit, crosstalk can approach and even drop below the crosstalk of the basic model with a binding site that is twice as long. This is a relevant comparison, because cooperative regulation does, in fact, have access to a total of 2*L* base pairs of recognition sequence. Furthermore, the optimal TF concentration C* required in the cooperative case is lower than in the single site case (Fig. 4d), making cooperativity a realistic crosstalk reduction mechanism.

The crucial assumption of the cooperative model presented above is that cooperative interaction between two TF molecules can only occur when they bind their cognate binding sites and never otherwise. This is a very restrictive assumption that is unlikely to hold in many documented models of cooperativity. For example, if cooperative interaction energy Δ originates in protein–protein interactions between the two TF molecules of the same species, this energy will plausibly be gained even when these same TF molecules come into contact while binding two nearby non-cognate sites. Similarly, synergistic activation^24^ or nucleosome-mediated cooperativity^41^ models also imply that noncognately-bound factors could contribute towards cooperativity, violating our assumption that cooperativity is exclusive to cognate binding.

To relax this assumption and study the effects of the resulting ‘non-cognate cooperativity', we recompute accordingly the crosstalk improvement relative to the basic model, as shown in Supplementary Fig. 18. Not surprisingly, we find that allowing cooperative interactions between TFs of the same type when bound non-cognately leads to much smaller reductions in crosstalk compared with cooperativity that is exclusive to cognate binding, as shown in Table 1. When non-cognate cooperativity is allowed, we can also look at the strong cooperativity (large Δ) limit and compare crosstalk improvement due to two TFs cooperatively binding two sites of length *L*, with the basic model of a single TF binding a site of length 2*L*. Now, cooperative regulation by two TFs is always inferior to the regulation by a single factor with a longer binding site (see Supplementary Note 5).

Dimerization of TFs is very common among prokaryotes, where TF monomers often dimerize in solution before binding to DNA. If the two binding sites in our model predominantly act as half-sites for the binding of a single dimer, the relevant equations for crosstalk are identical to non-cognate cooperativity in the large Δ limit, with *C* being the concentration of monomers. Thus, our theory is also applicable to this case, although dimerization in solution is often not considered a canonical example of cooperative regulation. Cooperative interactions conditional on DNA binding have been less frequently reported but are also known to occur in prokaryotes (for example, on proximal binding of two dimers); in experimentally documented cases, the interaction energies are weaker, Δ∼3 *k*_B_*T* (ref. 27), which still facilitates crosstalk reduction although it is accordingly smaller (Supplementary Fig. 18).

The two cases of cooperativity we considered here represent two extremes of a spectrum: cooperative interaction is either possible exclusively at the cognate site or at all sites equally. There probably exist intermediate situations, which help limit the occurrence of spurious cooperative interactions. A simple example of such a mechanism could use the positioning of the binding sites on the DNA: TF cooperative binding is limited only to pairs of sites, which are appropriately spaced. If different TF types use different spacing, the harmful effects of cooperativity at a particular non-cognate site pair will be restricted to a subset of TFs. More complex geometrical arrangements, for example, cooperative interactions involving DNA looping or allosteric effects between the two TFs and the DNA^42^, could provide similar benefits. Although possible in principle, these benefits should be considered as hypothetical, as direct experimental support for cooperativity that is exclusive to cognate binding is still lacking.

An important contribution to crosstalk is the erroneous activation of genes that should remain inactive. One might argue that any kind of global repression could alleviate this problem by preventing spurious transcription. We explored this scenario by extending our basic model to include an additional nonspecific repressor (Supplementary Note 6). Perhaps not surprisingly, we find that the minimal achievable crosstalk error in this extended scheme is exactly the same as in the basic setup, regardless of the concentration and the affinity of the sites.

**Figure 5:**
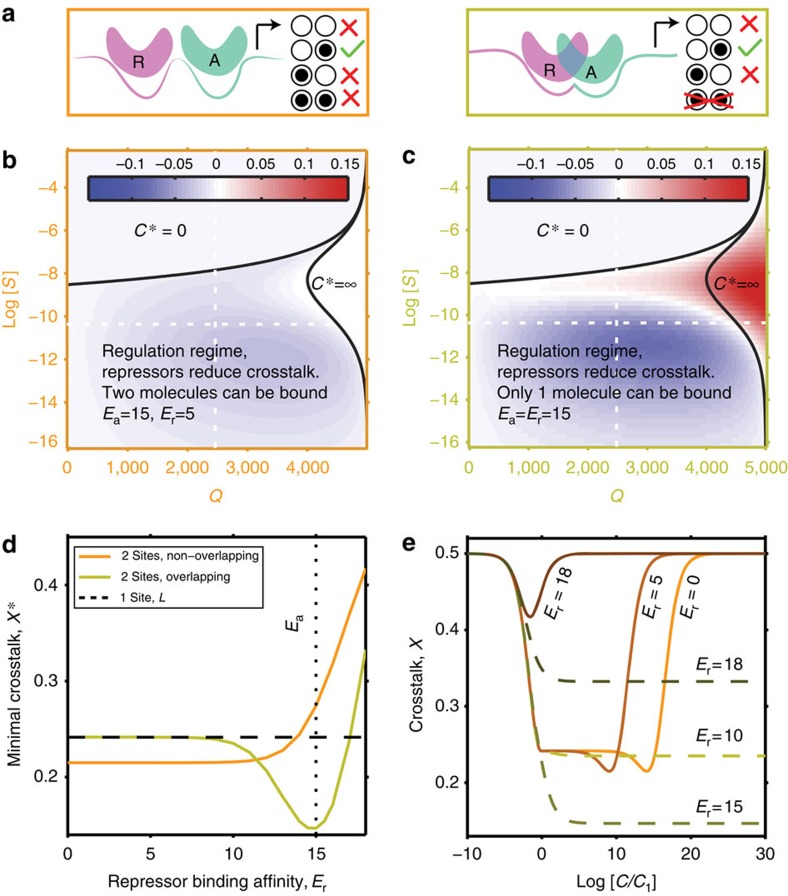
Combinatorial regulation by activators and repressors yields marginal improvements in crosstalk error. (**a**) Separate (left) or overlapping (right) binding sites for activators A and repressors R. A subset of binding configurations for cognate regulators is shown; transcription proceeds (green) only when the A site is bound by the cognate activator and the R site is unbound. (**b**,**c**) Difference (shown in colour) between minimal crosstalk achievable with activator–repressor regulation and the basic model of Fig. 3. With optimal value for the affinity of repressor sites (*E*_r_) selected in both cases, a small overall improvement in crosstalk error is seen in **b** and a larger improvement, but localized to log *S*≲−10, in **c**. At baseline parameters (white dashed lines), X*=0.2 for the non-overlapping case, X*=0.15 for the overlapping case and X*=0.23 in the basic model. (**d**) Dependence of the crosstalk on the repressor binding affinity *E*_r_ (activator affinity fixed at *E*_a_=15). When *E*_r_>*E*_a_, the crosstalk quickly increases: instead of helping prevent erroneous activation, repressors themselves bind too frequently in noncognate configurations, aggravating the crosstalk. For non-overlapping sites scenario, *E*_r_<<*E*_a_ is optimal, whereas in the overlapping sites case, *E*_r_=*E*_a_ is optimal. (**e**) Dependence of crosstalk on the total concentration, *C*, of transcription factors, for non-overlapping sites case (orange-brown curves representing different *E*_r_, as indicated) and overlapping sites case (green curves representing different *E*_r_ as indicated). The total concentration is optimally split between activators and repressors for each *C*, and is reported relative to the optimal concentration *C*_1_ of the basic model.

We next turned our attention to a sequence-specific repression mechanism. In an extension to our basic model, we equipped each gene with both an activator and a repressor site, such that each of these sites has its own cognate regulator (activator or repressor). For the *Q* genes that should be active, only their *Q* cognate activators (but not repressors) were present. For the remaining *M*−*Q* genes that should be inactive, only their cognate repressors (but not activators) were present. Repressor sites could have a different affinity (*E*_r_) than the activator sites (*E*_a_). To look for the minimal achievable crosstalk, we optimized over the concentration of activators, repressors and the affinity *E*_r_. Importantly, we considered two possible molecular arrangements on the promoter: in the non-overlapping sites scenario (Fig. 5a, left) the two binding sites could be occupied by regulatory molecules simultaneously, whereas in the overlapping sites scenario (Fig. 5a, right), either the activator or repressor site, but not both, could simultaneously be occupied. Whether this exclusion happens because the two binding sites literally overlap or due to more complex mechanisms is not crucial for our results. We assumed that a bound repressor inactivates transcription, regardless of the activator state; for a detailed list of molecular configurations on the promoter, see Supplementary Note 7.

In the non-overlapping case, small (∼10% at baseline parameters) decreases in crosstalk error are nominally possible, as shown in Fig. 5b. A detailed examination, however, argues against this mechanism for crosstalk reduction. Optimization in Fig. 5d assigns the repressor sites a very weak, or even vanishing, affinity for the TFs, *E*_r_<<*E*_a_: in essence, the repressor sites energetically favour staying empty to the same amount as binding a cognate repressor, to fight off non-cognate binding. As a costly consequence, the optimal concentration of the required TFs needs to be larger by an unreasonable factor, ∼20,000-fold, relative to the basic model, to achieve this small crosstalk reduction gain.

The overlapping case provides a greater crosstalk reduction (∼35% at baseline parameters), as shown in Fig. 5c. The optimal repressor sites have similar affinity to their cognate TFs as do the optimal activator sites, *E*_r_∼*E*_a_; the benefit of the repressors quickly vanishes if this condition is not met. The total required regulator concentration now no longer has a clearly defined optimum, but does exhibit a plateau where the crosstalk is minimized. Importantly, as shown in Fig. 5e, this plateau is reached for concentrations only somewhat higher than in the baseline case, making this solution biologically plausible.

In sum, the case for combinatorial regulation by activators and repressors is complicated. Combinatorial regulation provides a smaller absolute improvement than cooperativity, but this improvement is also centred around smaller values for binding site similarity, log (*S*)≲−10, where the crosstalk of the basic model is itself already lower. In contrast to our initial expectation, this small gain is realistically achievable only with one of the two regulatory schemes considered and only when its parameters are properly tuned.

Lastly, we considered the simplest AND-gate regulation scenario. The expression state of each gene is determined by the occupancy of two binding sites; in particular, activation is achieved by binding of a precisely specified, unique pair of cognate activating TFs. Crucially, in the ‘perfect combinatorial regulation' scenario, 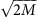 TF species (instead of *M*, as in the basic model) are sufficient to specifically regulate any subset of the *M* genes. As we show in Supplementary Note 8 and summarize in Table 1, this leads to a sizeable crosstalk reduction. Using 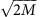 TF species means, on average, 
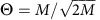
 regulated genes per TF. If sets of Θ genes were regulated jointly by a common TF, crosstalk should decrease as 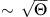, as we argued above. Supplementary Fig. 21 shows that for the AND-gate the decrease is somewhat smaller, but unlike in the simple scenario where each TF regulates groups of Θ genes with no possibility of control over individual genes, the AND-gate allows each gene to be regulated individually. Although this combinatorial strategy allows crosstalk reduction and has been documented at specific promoters, we point out that the predicted, square-root scaling of the number of TF species with the total number of genes, *M*, is inconsistent with published reports^43^,^44^, making it unlikely that crosstalk reduction is achieved through genome-scale combinatorial control as analysed here.

## Discussion

Finite specificity of recognition reactions is a fact of life at the molecular scale. In transcriptional regulation, which takes place in a mix of cognate and non-cognate TF species, the consequences of this fact could be severe—but have surprisingly not been taken to their logical conclusion so far. Here we constructed a theoretical framework for crosstalk that accounts for all possible cross-interactions between regulators and their binding sites. This global model enabled us to compute the lower bound on crosstalk and assess the effectiveness of various regulatory schemes. We derived limits to reliable gene regulation that depend only on the total number of genes *M*, the typical number of co-activated genes, *Q*, and the average level of similarity between pairs of binding sites, *S*.

We find that these parameters robustly define three possible regulatory regimes. A non-zero TF concentration that minimizes crosstalk exists only when binding sites are sufficiently distinguishable from each other and the typical number of co-activated genes is not extreme. We call this the ‘regulation regime.' The other two regimes are anomalous cases where regulation is dysfunctional. Looking closely at the boundaries between the three regulatory regimes, we find that the average similarity between binding sites, *S*, puts an upper bound to the total number of genes that an organism can effectively regulate^45^.

An analogous problem exists in protein–protein interaction networks, where protein function requires strong binding to a few partner proteins but avoidance of binding to all the others^6^,^7^. Previous works have studied the evolution of such networks by applying a combination of positive and negative design using computer simulations, concluding that ‘negative design' seriously constrains the possible architectures^6^,^46^,^47^,^48^. As a quantitative measure for the likelihood of specific versus nonspecific interactions, Johnson *et al*.^6^ used the minimal energy gap between specific and nonspecific interactions, in analogy to our measure of binding sites similarity *S*. They found a power-law scaling of the energy gap with the total number of proteins in the network and also found that it depends inversely on the size of binding surface, *L*—both results are in qualitative agreement with ours for the total number of genes *M* and length of the binding sites *L*. Similarly, a larger binding domain was found to enable a larger number of specific interactions in a protein mixture when other nonspecific interactions are excluded^47^. Johnson *et al*.^6^ also found that network designs in which some proteins have multiple specific partners (‘hubs') have higher crosstalk compared with networks with only pairwise interactions. At this point, protein–protein interaction networks significantly differ from TF–DNA interactions: if multiple binding sites share a common TF, these binding sites cannot bind each other, as would be the case for different protein species interacting with a common hub. Zhang *et al*.^7^ identified a trade-off between proteome diversity and concentration due to crosstalk considerations, concluding that the numbers found experimentally are close to the possible limit. Protein concentrations face trade-off: they should be high enough to form specific interactions, but not so high as to form many nonspecific ones. The optimal TF concentration in our model is determined by a similar trade-off. Analogous problems due to explosion of non-cognate configurations were studied in the context of prebiotic metabolism^49^ and the immune system, where receptors are selected to recognize foreign peptides, but avoid binding self-peptides^50^. In the context of TF–DNA interactions Sengupta *et al*.^21^ studied how the evolutionary mutation-selection balance tunes TF specificities to its DNA targets and how this depends on the number of targets. They identified a trade-off between avoiding the loss of current targets (for which a lower specificity is favoured) and avoiding the spurious recruitment of new ones (for which a higher specificity is favoured); they also report an inverse relation between the number of different targets and the TF specificity for each. An intriguing direction for future research is to explore how crosstalk might limit the complexity of regulatory networks in an evolutionary setting.

Where do real organisms find themselves in this parameter space? Prokaryotes tend to have longer binding sites and fewer genes than eukaryotes. In Table 2 we present typical biophysical parameters for each and the resulting crosstalk estimates. Although for prokaryotes we expect crosstalk to easily be between 1 and 10% even if each gene is regulated by a single site, and below 1% for biophysically realistic cooperative regulation, for eukaryotes the situation is significantly different. Even for a short genome of *M*=5,000 genes, such as yeast, or for longer genomes of metazoans where most of the genes have been non-transcriptionally silenced, we expect minimal crosstalk of X*=0.23. In an organism with *M*=20,000 regulated genes, crosstalk would increase substantially according to the basic model: >40% of all genes would be erroneously regulated. Incorporating known constraints on the biophysics of TF–DNA interaction (Supplementary Figs 16 and 17) increases crosstalk even further and pushes metazoan regulation towards the anomalous regime.

Complex regulatory schemes increase the specificity of gene regulation by cognate factors and high specificity was tacitly assumed to provide automatic resilience against crosstalk. In contrast, our analysis of several complex regulatory mechanisms reveals a more intricate picture. We focused on two broad classes of regulatory mechanisms. The first class comprises various schemes of cooperative regulation. Cooperativity can lower crosstalk, because it effectively increases the binding site length and energy and thus reduces binding site similarity. We found that the effectiveness of cooperativity for reducing crosstalk crucially depends on the strength of the cooperative interaction and on whether cooperative interactions are restricted exclusively to cognate sites. With respect to cooperative interaction strength, the optimal crosstalk reduction happens at very strong cooperativity, but this might be hard to realize biophysically. Commonly reported values are indeed small (3−5 *k*_B_*T*), comparable to the energetic contribution of only 1−2 bp in the TF–DNA interaction^27^,^28^. With respect to cooperative interactions being exclusive to cognate binding, such regulatory schemes, while optimal for crosstalk reduction, would require additional sequence recognition mechanisms and it is unclear to what extent they exist or how effective they are. If cooperative interactions can occur at non-cognate sites as well as is the case for most documented mechanisms of cooperativity, its effectiveness in mitigating crosstalk is significantly diminished. The second class of mechanisms we considered relies on combinatorial regulation by multiple TFs. As a representative example we studied combinatorial regulation by activators and repressors. Contrary to the common expectation that repression should eliminate spurious gene activation^24^,^25^, we found various mechanisms to be either ineffective (global repression) or providing marginal global improvement at best (activator–repressor regulation with overlapping binding sites). Although crosstalk can indeed be mitigated for particular gene(s) by employing a complex promoter architecture, this inevitably comes at a cost for the regulation of other genes. The intuitive explanation for the limited benefit of combinatorial schemes is that adding new regulatory components—in this case, repressors and their respective binding sites—drastically increases the number of possible non-cognate interactions, thereby potentially aggravating, instead of mitigating, the crosstalk problem. A similar detrimental effect due to growth in the number of undesired configurations with the number of molecular species has been reported in the study of molecular self-assembly^12^. A potentially powerful set of mechanisms are therefore schemes in which combinatorial regulation is used primarily to decrease the required number of molecular species, as in the simple AND-gate example we explored in Supplementary Note 8. Further work is needed to fully elucidate crosstalk limits in more general models of combinatorial control and cooperativity, with interesting parallels to precision in biochemical sensing, in equilibrium as well as out-of-equilibrium scenarios^3^,^29^,^51^,^52^.

An interesting result of our study is that various schemes of molecular control logic at promoters and enhancers^53^, while nearly equivalent in the absence of crosstalk, can behave very differently in the presence of non-cognate regulators^54^. For example, the issue of cooperative interactions during non-cognate binding is a striking demonstration of how a seemingly microscopic detail may influence global crosstalk, whereas it has no bearing on the aspects for which cooperativity has been studied traditionally: its ability to sharply activate the cognate gene in response to small increases in TF concentration. A similar remark applies for the case of overlapping versus non-overlapping binding sites in the combinatorial regulation scenario. By going beyond mean-field approximations, this could be extended to biologically relevant situations where pairs of binding sites overlap so as to share large sequence fragments^55^. Clearly, there is a need to further understand signal processing at complex promoters^56^ and for experimental measurements of crosstalk in various regulatory architectures.

Direct measurements of crosstalk are challenging, precisely because crosstalk is a global effect and experimentally influencing non-cognate binding in a controlled manner is difficult. An alternative approach would be to search for indirect signatures of crosstalk^57^. A promising line of research supported by a large body of recent experimental evidence would be to examine ‘pervasive transcription' in eukaryotes^15^,^58^ as a proxy for erroneous initiation, perhaps due to crosstalk interference.

Taken together, our findings suggest that global crosstalk represents a strong constraint in eukaryotic regulation that can be mitigated, but not easily removed. Initially, this conclusion was based on a greatly simplified model of gene regulation. We succeeded in relaxing many of our assumptions only to find that crosstalk constraints remain significant. This is because the major determinant of crosstalk is the binding site similarity *S*, which primarily depends on the typical mismatch energy 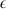 and the length of the binding sites, *L*. Although crosstalk could be reduced by extending binding site length and/or augmenting the binding energy, both parameters are severely constrained by a combination of biophysical and evolutionary factors. The scale of the mismatch energy is set by the energetics of hydrogen bonds to ∼2–4 *k*_B_*T*, whereas the length of individual binding sites in eukaryotes appears strongly constrained by evolutionary considerations to ∼10 bp^21^,^59^,^60^. Moreover, the performance of complex regulatory schemes, which appear beneficial at first glance, is also limited by the explosion of possible non-cognate configurations that may lead to erroneous regulation. These constraints should apply universally, beyond the specific mechanisms we analysed in detail: any regulatory scheme operating at equilibrium, no matter how complex, faces a fundamental limit to its achievable error, for reasons that led Hopfield^2^ to propose kinetic proofreading.

The main conclusion of our work is that crosstalk in gene regulation is far from being a solved problem. We find several commonly studied regulatory mechanisms to be insufficient for eliminating crosstalk in metazoans, at least when acting alone. Although it is theoretically possible that a combination of equilibrium mechanisms acting in unison could achieve low crosstalk levels, this possibility is by no means obvious and indeed appears unlikely. Alternatively, cells might have evolved out-of-equilibrium solutions where energy is deliberately spent to counteract the detrimental effects of crosstalk; example mechanisms could include permanent gene silencing, localization of transcriptional activity to specific cellular compartments or molecular reaction schemes for gene regulation that implement variants of kinetic proofreading^29^.

## Methods

We employ a thermodynamic model of regulation^23^,^27^,^61^, which postulates that the gene expression level depends on the equilibrium occupancy of TFs at the regulatory sites on the DNA. This model has been widely used to predict gene expression and has been experimentally validated in various systems^62^,^63^,^64^. In this framework, the binding probability of a TF to any binding site, cognate or non-cognate, is determined by two factors: the effective concentration of TFs and the binding energy.

We assume that the binding energy only depends on the number of mismatches between a particular binding site and the consensus sequence unique to the given TF. Each binding site can thus exist in either of the three possible states^61^: (i) bound by a cognate TF; (ii) bound by a non-cognate TF; or (iii) unbound. Binding of the cognate factor (i) is energetically the most favourable state and is assigned the energy *E*=0. The unbound state (iii) is usually energetically least favourable with energy *E*_a_>0. Between these two extremes there exist non-cognate-bound configurations (ii) with intermediate energies that depend only on the number of nucleotide mismatches *d* between the consensus sequence of the TF and the sequence of a given binding site, that, 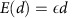, where 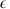 is the energy per mismatch. This mismatch energy model provides a tractable approximation to more detailed models^28^ and has been extensively used in the literature^20^,^39^.

In our model, the crosstalk error can be separated into two contributions that can be computed using basic statistical mechanics:

1. For a gene *i* that should be active and whose cognate TF is therefore present, error occurs if its binding site is bound by a non-cognate regulator (activation out of context due to crosstalk), or if the binding site is mistakenly unbound (gene is inactive). This happens with probability


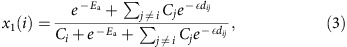


where *C*_*j*_ is the concentration of the *j*th TF, *d*_*ij*_ is the number of mismatches between the *j*th TF consensus sequence and the binding site of gene *i*, and 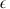 is the energy per mismatch; all energies are measured in units of *k*_B_*T*. Here we consider activation by a non-cognate TF as crosstalk; reasons for this choice, as well as an alternative model where such cross-activation is not considered an error state, are presented in Supplementary Note 9.

2. For a gene *i* that should be inactive and whose cognate TF is therefore absent, crosstalk error only happens if its binding site is bound by a non-cognate regulator (erroneous activation) rather than remaining unbound. This happens with probability


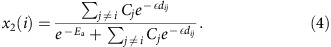


We define the global crosstalk *X* as the expected fraction of erroneously regulated genes. In our basic model where all genes are identically regulated and TFs for genes that need to be activated are present at equal concentrations (that is, *C*_*j*_=*C*/*Q*, where *C* is the total concentration of all TFs and *Q* is the number of distinct TF species present simultaneously), we show in Supplementary Note 1 that the crosstalk is


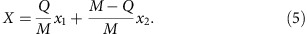


Global crosstalk *X* ranges between zero (no erroneous regulation) and one (every gene is mis-regulated).

Further methods are described in Supplementary Notes 1–9.

### Data availability

The authors declare that all data supporting the findings of this study are available within the article and its Supplementary Information file.

## Additional information

**How to cite this article:** Friedlander, T. *et al*. Intrinsic limits to gene regulation by global crosstalk. *Nat. Commun.* 7:12307 doi: 10.1038/ncomms12307 (2016).

## Supplementary Material

Supplementary Information Supplementary Notes 1-9, Supplementary References, Supplementary Figures 1-23 and Supplementary Tables 1-5

## Figures and Tables

**Table 1:** Comparison of crosstalk levels between the different variants of the model.

**Model**	**Crosstalk (at baseline parameters)**	**Remarks**
Basic model (activators-only)	0.23	
Basic model (repressors-only)	0.23	
Mixed model (activators+repressors)	0.14	2,000 genes expressed in 20% of the env., 3,000 genes in 70%
Genes of unequal importance	0.31	10% of the genes are important and penalized 10 × the ‘normal' rate
		The resulting error per important gene decreases to 0.1, but for the other genes increases to 0.33.
Unequal weights for the two error types	0.17	*b*=0.5, weight of erroneously active genes is half that of genes that are erroneously inactive
Each TF regulates exactly Θ=10 genes	0.08	Also holds for *P*(Θ)∼Poisson 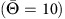
Activators+global nonspecific repressor	0.23	Cannot reduce crosstalk
Activators+specific repressors (non-overlapping)	0.2	
Activators+specific repressors (overlapping)	0.15	
Perfect AND-gate combinatorial regulation	0.07	Uses only 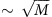 TF species
Generic cooperativity	0.064	For example, dimerization, direct TF–TF contacts, TF/nucleosome competition and so on. Two bindings sites, each of length *L*=10
Cooperativity exclusive to cognate binding	0.006	Currently unknown molecular mechanisms, two bindings sites, each of length *L*=10.

Baseline parameters are: *Q*=2,500, *M*=5,000, log(*S*)=−10.5—equivalent to a model where binding sites for distinct TFs are different from each other in at least 2 bp (*d*_min_=2) with binding sites of *L*=10 bp and binding energy 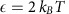 per mismatch.

**Table 2:** Comparison of relevant parameters and crosstalk values between prokaryotes and eukaryotes.

	**Prokaryotes**	**Eukaryotes**
Binding site length	10–20 bp	6–10 bp
Binding site similarity, *S*	−20≲log (*S*)≲−13	−15≲log (*S*)≲−9
Number of genes, *M*	A few thousands	5,000–20,000
Crosstalk in the basic model	1–10%	20–50% (Depending on *M*)
Crosstalk with cooperative regulation	<1%	∼10%
