# Supplementary material for: Intrinsic limits to gene regulation by global crosstalk
Source: Nat Commun. 2016 Aug 4;7:12307. doi: 10.1038/ncomms12307 (PMC4976215; doi:10.1038/ncomms12307)
Supplement: Supplementary Information — Supplementary Notes 1-9, Supplementary References, Supplementary Figures 1-23 and Supplementary Tables 1-5 [file ncomms12307-s1.pdf]

Tamar Friedlander, Roshan Prizak, Călin C. Guet, Nicholas H. Barton and Gašper Tkačik

Supplementary Note 1 Basic model – analytical solution

We assume that the genome of a cell contains M "target" genes, each of which is regulated by a single unique transcription factor binding site (BS). In the basic formulation, there exist also M distinct TF types, such that each TF can preferentially activate its corresponding target gene by binding to its binding site. At any point in time, however, not all M TF types are present: we assume that only subsets of size $Q \leq M$ are present at some nonzero concentration, and that the optimal gene regulatory state for the cell would be to express exactly and only those genes for which the Q corresponding TFs are present.

Let regulation be determined by the (mis)match between the binding site sequence and the recognition sequence of any transcription factor. Each binding site is associated with a single TF type with which it forms a perfect match – this is the cognate TF for the given binding site. However, each site could also occasionally be bound by other (noncognate) TFs, at an energetic cost of a certain number of mismatches. Following earlier works [1, 2], we assume that the contribution of mismatches at individual positions in a binding site to the binding energy is equal, additive, and independent. We define the energy scale such that binding with cognate TF has zero energy and all other binding configurations have positive energies, proportional to the number of mismatches d, $E = \epsilon d$, where ϵ is the per-nucleotide binding energy. The unbound state has energy E_a with respect to the cognate bound state. The different states and their energies are illustrated in Fig. 3A in the main text. We employ a thermodynamic model to calculate the equilibrium binding probabilities of cognate and noncognate factors to each binding sequence.

TFs can also be non-specifically bound to the DNA. These configurations only sequester TFs from free solution, but do not directly interfere with gene expression. As explained later, we will lump together the TFs freely diffusing in the solution, as well as nonspecifically bound TFs and any other TF "reservoirs" into one effective concentration of available TFs (equivalently, we work with the chemical potential of the available TFs using the grand-canonical ensemble).

Previous studies calculated the probability of a given transcription factor to be bound or unbound to certain DNA sequences [2]. These probabilities were calculated assuming that the site is vacant or bound by the TF under study, but not bound by TFs of other types. This approach is cumbersome when a large number of TF types are considered simultaneously, because the probability that the site is bound by other factors is non-negligible, and due to steric hinderance, a site cannot be bound by more than one molecule at any given time. Previous studies also proceeded by using the canonical ensemble. These two modeling choices together make the problem of many TFs binding to multiple binding sites coupled and not easily tractable, because one would need to enumerate all possible combinations of TF-BS states. However, an alternative and much simpler approach is to employ the grand-canonical ensemble, and calculate the binding probabilities for the binding sites, rather than for the TFs. The necessary assumption is that binding sites behave independently (e.g., they are sufficiently separated on the DNA so that binding at one site does not overlap the binding at another, or if it does, this is treated explicitly). Underlying the

grand-canonical ensemble is the assumption that TFs are present at sufficient copy numbers, so that the binding of a single site under consideration does not appreciably affect the chemical potential of the remaining TFs. Experimental support for such decoupling and the applicability of the grand-canonical approach has been demonstrated recently [3]. In the following we assume equal concentrations of all TF types.

We distinguish two contributions to crosstalk:

1. For a gene i that should be active and whose cognate TF is therefore present, error occurs if its binding site is bound by a noncognate regulator (activation out of context due to crosstalk), or if the binding site is unbound (gene is inactive). This happens with probability

$$x_1^i(\{C_j\}) = \frac{e^{-E_a} + \sum_{j \neq i} C_j e^{-\epsilon d_{ij}}}{C_i + e^{-E_a} + \sum_{j \neq i} C_j e^{-\epsilon d_{ij}}}, \tag{S1}$$

   where C_j is the concentration of the jth TF, d_{ij} is the number of mismatches between the jth TF consensus sequence and the binding site of gene i, ϵ the energy per mismatch and E_a the energy difference between unbound and cognate bound states; all energies are measured in units of $k_B T$.

2. For a gene i that should be inactive and whose cognate TF is therefore absent, crosstalk error only happens if its binding site is bound by a noncognate regulator (erroneous activation) rather than remaining unbound. This happens with probability

$$x_2^i(\{C_j\}) = \frac{\sum_{j \neq i} C_j e^{-\epsilon d_{ij}}}{e^{-E_a} + \sum_{j \neq i} C_j e^{-\epsilon d_{ij}}}. \tag{S2}$$

In general $x_{1,2}$ depend on the specific set of pair-wise distances d_{ij} between the consensus sequence of each TF present and the site of gene i. Hence they could vary between genes, and even for each gene different sets of TFs can yield different values of crosstalk. In the following we assume a fully symmetric setup, such that all genes are equivalent in their sensitivity to crosstalk ($x_{1,2}$ is independent of i). We assume that for each gene the mismatches d_{ij} of all the noncognate TFs are distributed according to a probability density $p(d)$ (independent of the gene). For a particular gene i, clearly different sets of TFs provide different pairwise distances d_{ij}. However, for $Q \gg 1$ the fraction of sets of same size Q that yield distances which are distributed very differently from $p(d)$ is small. In the following we neglect this fraction and assume that all choices of Q TFs yield exactly the same crosstalk contribution $x_{1,2}(Q, M)$; this mean-field assumption is explicitly validated by numerical simulations in Supplementary Note 3. We will also consider that all possible sets of Q TFs (sets of genes that need to be active) are equally likely to occur. See Supplementary Note 9 for the alternative definitions of x_1 and x_2.

Our next step is to calculate total crosstalk as a function of the above parameters (the total number of binding sites M and the number of TF types available at any given time Q). We define total crosstalk as the *fraction* of genes found in any of the possible erroneous states. We assume that the particular choice of Q TFs that are present is random (hence we average over all possible ways to choose Q out of M TFs). In reality only certain sets of TFs need to be active together in which case the genes that are co-activated could have mutually similar binding sites, especially if they were regulated by the same TF, compared to genes that are activated separately, possibly by different TFs. In Supplementary Note 1 we treat a simple extension of our model where each TF can co-regulate several target genes. We also assume equivalence between the two types of error (we relax this assumption below).

Clearly, if each of the Q genes that should be active has probability x_1 to be in any of the crosstalk states, then the *expected number* of genes in that state is Qx_1. Similarly, of the genes that should be inactive the *expected number* that are in crosstalk state is $(M-Q)x_2$. To obtain the *fraction* of genes in any of the crosstalk states we simply divide by the total number of genes M:

$$X(Q, M, x_1, x_2) = x_1 \frac{Q}{M} + x_2 \frac{M-Q}{M}. \tag{S3}$$

Using the definition of S introduced in the main text

$$\sum_{j \neq i} C_j e^{-\epsilon d_{ij}} = \frac{C}{Q}(Q-1) \sum_d P(d) e^{-\epsilon d} \approx C \sum_d P(d) e^{-\epsilon d} \equiv C S_i(\epsilon, L), \tag{S4}$$

where we approximated $Q - 1 \approx Q$ which is valid for $Q \gg 1$ (an assumption we make here and throughout the paper). $S(\epsilon, L)$ is an average similarity measure between all pairs of binding sites. If binding site sequences are drawn randomly from a uniform distribution, $S = (\frac{1}{4} + \frac{3}{4}e^{-\epsilon})^L$. This is easy to derive: since individual base pairs are assumed to be statistically independent, at each position the probability of a random sequence to be identical to a given TF consensus sequence is $1/4$, whereas with probability $3/4$ it is different, implying a decrease of $e^{-\epsilon}$ in binding energy. Since the complete binding site consists of L independent base pairs, this expression for a single base pair is now raised to the power of L.

The expressions for $x_{1,2}$ read:

$$x_1 = \frac{e^{-E_a} + CS}{\frac{C}{Q} + e^{-E_a} + CS} \tag{S5a}$$

$$x_2 = \frac{CS}{e^{-E_a} + CS}. \tag{S5b}$$

The two extreme cases occur when TF concentrations are either zero or very large (Table 1). If $C = 0$, $x_1 = 1$ and $x_2 = 0$, i.e., x_1 is maximal due to binding sites that should be bound, while zero error for x_2 occurs due to binding sites that should be unbound. The total error then amounts to the fraction of genes that need to be activated $X(C = 0) = Q/M$. At the other extreme, if $C \to \infty$, $x_1 = SQ/(1 + SQ))$ and $x_2 \approx 1$, i.e., no site is left unbound. The magnitude of x_1 error due to noncognate binding is determined by the binding site similarity S. If $QS \ll 1$, $x_1 \approx QS - (QS)^2$. The total crosstalk then amounts to $X(C \to \infty) = 1 - \frac{Q/M}{1+SQ}$. If $SQ \ll 1$, $X \approx 1 - \frac{Q}{M}(1 - SQ)$.

Next, we analyze the dependence of crosstalk on various parameters. One unknown in these expressions is the TF concentration C. Because we are searching for a lower bound on crosstalk, we can find the concentration that minimizes X. Taking the derivative of X and solving for its zeros,

$$\frac{\partial}{\partial C} X(Q, M, x_1, x_2) = 0,$$

we find two potential extrema

$$C^*_{1,2} = \frac{Q e^{-E_a} \left(S(SMQ - Q(SQ+2) + M) \pm \sqrt{S(M-Q)} \right)}{S\left(-M(SQ+1)^2 + SQ^2(SQ+3) + Q\right)},$$

	x_1	x_2	crosstalk, X
	$\frac{e^{-E_a}+CS}{\frac{C}{Q}+e^{-E_a}+CS}$	$\frac{CS}{e^{-E_a}+CS}$	$\frac{Q}{M}x_1+\frac{M-Q}{M}x_2$
$C=0$	1	0	Q/M
$C=\infty$	$\frac{SQ}{1+SQ}$	1	$1-\frac{Q/M}{1+SQ}$
optimal C; only activators	$\frac{1+QZ}{1+Z/S+QZ}$	$\frac{QZ}{1+QZ}$	$\frac{Q}{M}\frac{1+QZ}{1+Z/S+QZ}+\frac{M-Q}{M}\frac{QZ}{1+QZ}$
optimal C; activators and global repressor	$\frac{1+QZ}{1+Z/S+QZ}$	$\frac{QZ}{1+QZ}$	$\frac{Q}{M}\frac{1+QZ}{1+Z/S+QZ}+\frac{M-Q}{M}\frac{QZ}{1+QZ}$

Supplementary Table 1: Crosstalk errors in the basic model. Per-gene errors of the two types: x_1 is the error of a site whose cognate TF exists and the site should therefore be bound, but is either unbound or bound by a noncognate factor. x_2 is the error of a site whose cognate factor does not exist, and the site should therefore be unbound, but is bound by a noncognate factor. The last column shows the total crosstalk, averaged over all M sites.

but only one of them can yield non-negative concentration values (and is consistently a minimum):

$$C^* = \frac{Qe^{-E_a}\left(S(SMQ-Q(SQ+2)+M)-\sqrt{S(M-Q)}\right)}{S\left(-M(SQ+1)^2+SQ^2(SQ+3)+Q\right)}. \tag{S6}$$

For small S the leading terms in the optimal concentration are

$$C^* = \frac{e^{-E_a}Q}{\sqrt{S(M-Q)}} - \frac{e^{-E_a}Q(M-2Q)}{M-Q} - \frac{e^{-E_a}Q^2(2M-3Q)\sqrt{S}^{3/2}}{M-Q} + O[S]. \tag{S7}$$

Substituting Eq. (S6) back into Eq. (S3) yields the minimal achievable crosstalk:

$$X^* = \frac{Q}{M}\left(-S(M-Q)+2\sqrt{S(M-Q)}\right). \tag{S8}$$

For a constant number of co-activated genes Q, X^* increases to leading order like the square root of S,

$$X^* = \frac{2Q\sqrt{M-Q}}{M}\sqrt{S}+O[S]. \tag{S9}$$

Substituting C^* into the single gene crosstalk expressions Eqs. (S1)-(S2), we obtain the minimal per-gene crosstalk

$$x_1^* = \sqrt{S(M-Q)} \tag{S10a}$$

$$x_2^* = SQ\left(\frac{1}{\sqrt{S(M-Q)}}-1\right). \tag{S10b}$$

Since crosstalk must be in the range [0,1] and $M \geq Q$, this solution is only valid under the condition that $S(M-Q)<1$. Thus, minimal crosstalk has 3 regimes:

1. For $S > 1/(M - Q)$, crosstalk is minimized by taking $C = 0$. This is the "no regulation" regime. In this case, crosstalk amounts to Q/M, which is simply the fraction of genes that were supposed to be activated (but are not due to lack of their TFs).

2. For $Q > Q_{\max}(S, M)$, crosstalk is minimized by taking $C \to \infty$; this is the "constitutive regime." $Q_{\max}(S, M)$ is given by two of the roots of the 4th order equation, $S(M + SMQ - 2Q - SQ^2) - \sqrt{S(M-Q)} = 0$, solved for Q. We find the boundaries between the 3 different regulatory regimes by solving for $C^*(S, M, Q) = 0$.

3. Otherwise, there is an optimal concentration $0 < C^* < \infty$, given by Eq. (S6), that minimizes crosstalk; this is the "regulation regime."

The boundary between the first and third region is at $S^* = \frac{1}{M-Q}$ and the boundary between the second and the third is at $S^* = \frac{-2M+3Q\pm\sqrt{Q(5Q-4M)}}{2Q(M-Q)}$. Hence, the second region (where $C^* = \infty$) only applies for $Q > \frac{4M}{5}$. Fig. 2(b) illustrates the dependence of the TF concentration C^*, which minimizes crosstalk, on the number of co-activated genes Q. It demonstrates how the range in which $0 < C^* < \infty$ gets narrower when S increases. Fig. 1 demonstrates crosstalk and C^* values for $M = 20,000$ (compare to Fig. 3 in the main text with $M = 5000$).

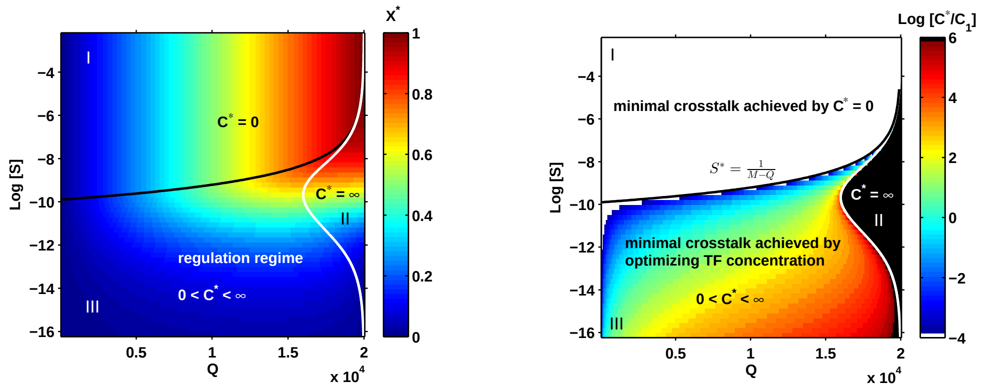

(a) (b)

Supplementary Figure 1: **Crosstalk in the basic model for** $M = 20,000$. Panel (a) shows the minimal crosstalk, X^*; panel (b) shows the optimal TF concentration, C^*. These results are analogous to Fig. 3 of the main paper, which is computed for $M = 5000$. The results for two different M are qualitatively similar and show 3 different regimes of regulation. We make the following observations: **(i)** for larger M, the $C^* = 0$ regime expands to include lower S values, as expected from the analytical solution for the regime boundaries; **(ii)** if the fraction of co-activated genes, Q/M, remains constant, the crosstalk *increases* with M, as it also depends on the absolute number of inactive genes $M - Q$ (see Eq. (S8)). The discrepancies at small Q between the black solid curve separating the "no regulation" and "regulation" regimes, and the numerically computed C^* values are due to the approximation $Q - 1 \approx Q$.

Basic model: Dependence on variables

Dependence on TF concentration

The optimal TF concentration C^* in our model arises as a trade-off between the Q genes that need to be active (for which a higher C is favored) and the $M - Q$ genes that need to be inactive (for which

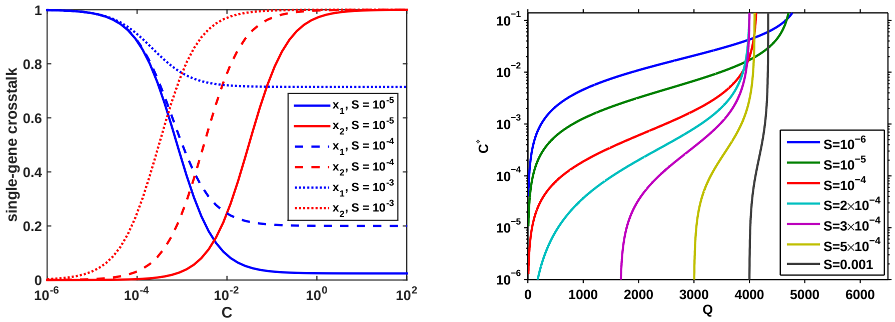

(a) (b)

Supplementary Figure 2: **How is optimal TF concentration C^* determined?** (a) x_1 crosstalk component (genes that should be active) decreases with TF concentration C, whereas x_2 crosstalk component (genes that should remain inactive) shows the opposite trend. Curves of x_1 and x_2 (crosstalk of a single gene) vs. C are illustrated for various values of S. While x_2 can be fully eliminated if $C = 0$, x_1 has a residual component which depends on S even for infinite C. Both crosstalk types increase with the similarity between the binding sites S (compare curves with various S values). (b) The optimal concentration C^* is a decreasing function of the similarity S for all Q values. At fixed M, the optimal TF concentration, C^*, diverges with the number of co-activated genes, Q. This leads to the "constitutive regime," where crosstalk is mathematically minimized by taking $C = \infty$. Shown is the optimal concentration C^* as a function of the number of co-activated genes Q, for various S values; M is fixed at 5000. The value of Q at which C diverges depends on S. For small Q, we require $M - 1/S < Q$, otherwise the optimal concentration is in the $C^* = 0$ regime. For the lower S values crosstalk can be minimized for $0 < Q < Q_{\text{max}} < M$, whereas for higher S values there exists also a value for Q_{min}, such that $0 < Q_{\text{min}} < Q < Q_{\text{max}} < M$. In other words, higher S leads to a narrower range of Q where the crosstalk can be effectively minimized.

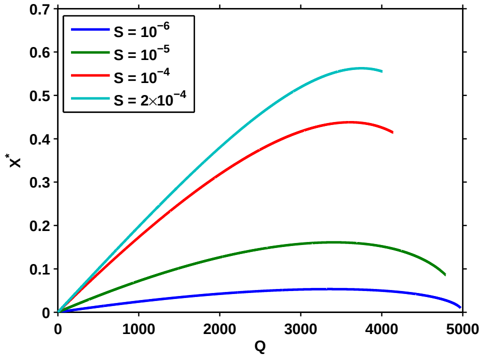

Supplementary Figure 3: **Minimal crosstalk X^* is an increasing function of the similarity S and has a non-monotonous dependence on the number of active genes Q.** The balance between genes that need to be active (x_1 crosstalk type) and genes that need to remain inactive (x_2 crosstalk type) causes a non-monotonous dependence of the *total crosstalk* on the number of active genes Q, which has a maximum at an intermediate Q value. Curves are shown only in the regulation regime, where crosstalk is minimized by a finite TF concentration. The curves are truncated at the point of transition to regime II where TF concentration formally diverges to infinity.

a lower C is favored). Note, however, the asymmetry between the two crosstalk types: while the x_2 component (genes that should remain inactive) can be completely suppressed by having no TF ($C = 0$), the opposite does not hold. The x_1 component (genes that should be active) cannot

be fully eliminated even for infinitely high C, because of the cross-activation between the distinct genes that should be active; see Fig. 2(a). This trade-off varies with the relative weights of x_1 and x_2, which depend on both Q and S. We find that a concentration C^* that minimizes crosstalk exists only in the third regime ("regulation regime"). In the first regime where $S < 1/(M-Q)$, binding sites are so similar that crosstalk due to the inactive $M-Q$ genes dominates the total crosstalk. Hence the choice of $C^* = 0$ completely eliminates x_2 crosstalk, and minimizes the total crosstalk. In the second regime, where a large number of genes Q need to be active, crosstalk due to the Q active genes dominates (x_1 type), hence C^* diverges to infinity. Fig. 2(b) illustrates curves of the optimal concentration C^* as a function of the number of active genes Q for constant values of S. As Q increases, the relative weight of the genes that need to be active increases, hence C^* is always a monotonously increasing function of Q.

Dependence on the similarity S

Both crosstalk types x_1 and x_2 increase with the similarity S (see Fig. 2(a)). For a fixed Q, C^* *decreases* as a function of S. Again, this is because for larger S the weight of the genes that should remain inactive is more significant, hence the trade-off shifts towards lower TF concentrations (but the minimal crosstalk X^* still increases!). This behavior applies only in the regulation regime, hence for $M - \frac{1}{S} < Q < Q_{\text{max}}$. For larger values of Q ($Q > Q_{\text{max}}$), a more complex behavior is found because by changing S we pass through all three regimes: C^* then first decreases, then diverges (because it enters the second regime), but then decreases back again.

Dependence on the number of active genes Q

The two crosstalk types show opposite dependence on the number of active genes Q: crosstalk per gene that needs to be active (x_1) decreases with Q, whereas crosstalk per gene that needs to remain inactive increases with Q. The total crosstalk is a weighted sum of both with varying weights, hence it is not surprising that the *total crosstalk* has a non-monotonous dependence on the number of active genes Q with a maximum at an intermediate value; see Fig. 3. The optimal TF concentration C^* increases with the number of active genes Q; see Fig. 2(b).

Basic model with regulation by repressors only

Our basic model assumed that all gene regulation is achieved by using specific activators to drive the expression of genes that would otherwise remain inactive. An alternative formulation of the problem postulates that genes are strongly expressed without TFs bound to their regulatory sites, but need to be repressed by the binding of specific regulators to stop their expression. Indeed, many bacterial genes seem to be regulated in this way. We thus studied this complementary model, in which all regulators are repressors instead of activators. We assume, as before, that Q out of M genes should be active, but now this implies that $M-Q$ types of cognate repressors are present for all the genes that should remain inactive.

The expressions for crosstalk per gene that should be active (x_1) or inactive (x_2) read:

$$x_1 = \frac{CS}{e^{-E_a} + CS} \tag{S11a}$$

$$x_2 = \frac{e^{-E_a} + CS}{\frac{C}{M-Q} + e^{-E_a} + CS}. \tag{S11b}$$

The total crosstalk is still

$$X = \frac{Q}{M}x_1 + \frac{M-Q}{M}x_2. \tag{S12}$$

Eqs. (S11) are mathematically identical to Eqs. (S5), where the roles of Q and $M-Q$ are simply swapped. Not surprisingly, the minimal crosstalk in this case is:

$$x_1^* = \frac{(M-Q)S(1-QS)}{QS+\sqrt{QS}} \tag{S13a}$$

$$x_2^* = \sqrt{QS} \tag{S13b}$$

$$X^* = \frac{M-Q}{M}(2\sqrt{QS} - QS), \tag{S13c}$$

which is valid for $S < 1/Q$.

The optimal TF concentration that minimizes crosstalk is now

$$C^* = \frac{e^{-E_a}(M-Q)(1-QS)}{\sqrt{QS}+QS(2-QS)+MS(QS-1)}. \tag{S14}$$

The minimal crosstalk and optimal concentration are illustrated in Fig. 4. It retains the 3 regulatory regimes observed with activators only:

1. For $S > 1/Q$ we obtain the "no regulation" regime where crosstalk is minimized by taking $C = 0$.
2. For $Q < Q_{\text{min}}(S, M)$ we obtain the "constitutive regime" where crosstalk is minimized by taking $C \to \infty$. Q_{min} is obtained when C^* of Eq. (S14) diverges (the denominator equals to zero).
3. Otherwise, there is an optimal concentration $0 < C^* < \infty$, given by Eq. (S14), that minimizes crosstalk; this is the "regulation regime."

The three regions are marked with Roman numerals, in accordance with Fig. 3 of the main text. The boundaries between the three regimes are now: $S^* = 1/Q$ (between regimes I and III) and $S^* = \frac{M-3Q\pm\sqrt{(M-Q)(M-5Q)}}{2Q(M-Q)}$ (between regime II to both I and III).

The results are clearly a mirror image of the results shown in Fig. 3 of the main text for the activator-only basic model. They can be obtained simply by mapping $Q \to M - Q$. Since we keep the convention that Q is the number of genes that are active, the difference in regulation strategies amounts to having either Q activator types and keeping $M - Q$ binding sites unbound (activator-only) or having $M - Q$ repressor types and keeping Q binding sites unbound. Comparing the expressions for minimal crosstalk, Eq. (S13c) to Eq. (S8), we conclude that crosstalk depends on the *fraction* of TFs that are expressed and on the *absolute number* of binding sites that need to remain unbound.

Breaking the symmetry between the two crosstalk types

In our basic model we made a simplifying assumption that the two crosstalk types, x_1 and x_2, have equal weights: not activating a gene that should be active or erroneously activating a gene that

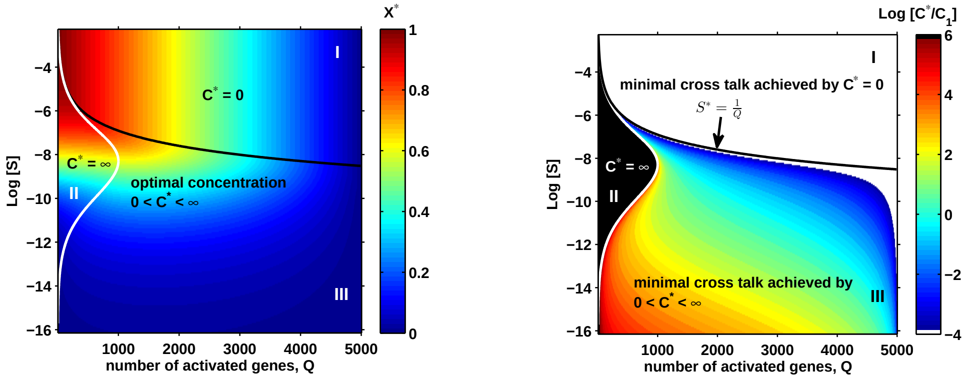

(a) (b)

Supplementary Figure 4: **Crosstalk in the basic model with regulation by repressors alone is a mirror image of regulation with activators only**. Panel (a) shows the minimal crosstalk, X^*; panel (b) shows the optimal TF concentration, C^*. These results are analogous to Fig. 3 of the main paper, which is computed for regulation with activators only. The observed picture is an exact mirror image of Fig. 3 of the main text, namely Q maps to $M - Q$, where we keep the convention that Q denotes the number of genes that should active. The difference is that in the activator-model activating Q genes requires Q types of activators, whereas in the repressor model this requires $M - Q$ types of repressors.

should be silenced are assumed to be equally disadvantageous. We now relax this symmetry by allowing different weights, a and b, for the two crosstalk types, to model possible differences in their biological significance. Eq. (S3) for the total crosstalk now takes the form:

$$X = a\frac{Q}{M}x_1 + b\frac{M-Q}{M}x_2. \tag{S15}$$

The expression for the optimal TF concentration then reads:

$$C^*(a,b) = \frac{e^{-E_a}Q(\pm\sqrt{abS(M-Q)} - S(aQ - b(M-Q)(1+SQ)))}{S(aSQ^2 - b(M-Q)(1+SQ)^2)}, \tag{S16}$$

where again only one of the two solutions yields non-negative concentration values. The resulting minimal crosstalk is:

$$X^*(a,b) = \frac{Q}{M}(-Sb(M-Q) + 2\sqrt{abS(M-Q)}). \tag{S17}$$

Setting $a = b = 1$ reduces the above formula to the previous solution, Eqs. (S6)-(S8). Note the asymmetry between the two crosstalk types: if $b = 0$, i.e., when crosstalk in genes that should remain inactive is insignificant, the minimal achievable crosstalk equals zero. This is not true in the other extreme case, when $a = 0$. In Fig. 5 we show that the three different regulatory regimes still exist under this generalized definition of crosstalk, but their boundaries may shift.

Breaking the symmetry between the co-activated genes

In our basic model we imposed full symmetry between the Q co-activated genes: they contributed equally to crosstalk and all Q types of TFs were assumed to exist in equal concentrations. We now

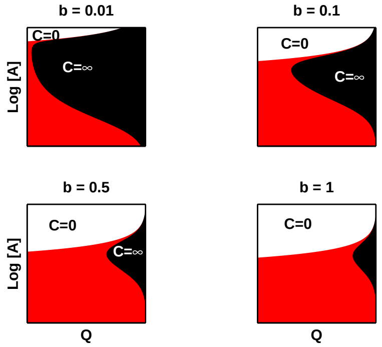

Supplementary Figure 5: **The three different regulatory regimes robustly exist even if the relative weight of the two crosstalk types vary.** To break the symmetry between the two error types we consider a redefined crosstalk, $X(b) = \frac{Q}{M}x_1 + b\frac{M-Q}{M}x_2$ (in the basic model $b = 1$). For different values of b (the cost of mis-activating genes that should remain inactive), all three regulatory regimes are preserved, although their boundaries shift. The weight of the first crosstalk type (mis-regulating genes that should be active) is equal in all cases. Red shows the "regulation regime," ($0 < C^* < \infty$). As erroneous activation is penalized less (decreasing b), the "no regulation" ($C^* = 0$, white) regime shrinks, whereas the constitutive expression regime ($C^* = \infty$, black) expands, as expected.

relax these assumptions. We examine the situation in which a fraction h of these Q genes is more important to the functioning of the cell. Mathematically, we postulate that the per-gene crosstalk error for the important genes contributes with a γ-times higher weight to the total crosstalk relative to the non-important genes. We introduce an additional degree of freedom to the model, by allowing the concentration of the TFs to split unevenly between important and other genes: each important gene has TFs present at concentration C_0, while a TF of a non-important gene is present at concentration $C_0 = \eta C_1$.

As $hQC_0 + (1-h)QC_1 = C$ we obtain:

$$C_1 = \frac{C}{Q}\frac{1}{(1-h+h\eta)} \tag{S18a}$$

$$C_0 = \eta C_1 = \frac{C}{Q}\frac{\eta}{(1-h+h\eta)} \tag{S18b}$$

If either $h = 0$ or $\eta = 1$ this reduces back to the basic model with $C_0 = C_1 = C/Q$. The total crosstalk now takes the form:

$$X = \gamma h\frac{Q}{M}x_0 + (1-h)\frac{Q}{M}x_1 + \frac{M-Q}{M}x_2 \tag{S19a}$$

$$x_0 = \frac{e^{-E_a} + CS\left(1 - \frac{\eta}{Q(1+h(\eta-1))}\right)}{e^{-E_a} + \frac{\eta C/Q}{1+h(\eta-1)} + CS\left(1 - \frac{\eta}{Q(1+h(\eta-1))}\right)} \tag{S19b}$$

$$x_1 = \frac{e^{-E_a} + CS\left(1 - \frac{1}{Q(1+h(\eta-1))}\right)}{e^{-E_a} + \frac{C/Q}{1+h(\eta-1)} + CS\left(1 - \frac{1}{Q(1+h(\eta-1))}\right)} \tag{S19c}$$

$$x_2 = \frac{CS}{e^{-E_a} + CS}, \tag{S19d}$$

where x_0 is the per-gene error of the important genes, x_1 is the error of other genes that need to be

activated, and x_2, as before, denotes crosstalk at genes that need to be kept inactive.

We can optimize numerically for both the total TF concentration C and the factor η by which the TF concentration of the important genes is amplified. Alternatively, we can assume that C remains fixed at the optimal value for the case where all genes are equally important, and only optimize for η. We display the latter option in Fig. 6, to explore crosstalk at varying h under equal resource constraints.

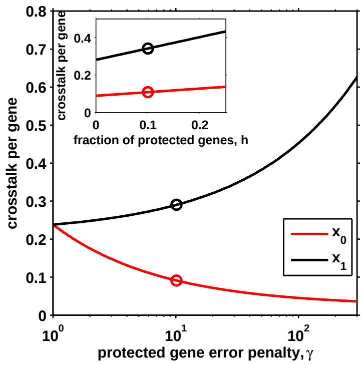

Supplementary Figure 6: **Crosstalk can be reduced for a subset of important genes at the cost of increasing the total crosstalk.** To break the symmetry between genes, we define a fraction h (out of Q) genes as important, having γ-times higher contribution to the total crosstalk. TF concentration for these genes is optimized separately, subject to the total TF concentration C remaining fixed to its optimal value in the symmetric, $\gamma = 1$, case. We show the crosstalk per important gene, x_0 (red), and per a normal gene, x_1 (black), as a function of γ (for $h = 0.1$). The inset shows the same as a function of h (for $\gamma = 10$). Per-gene crosstalk increases approximately linearly with h and important genes achieve $\sim \sqrt{\gamma}$ smaller crosstalk relative to normal genes.

The special case when only a single gene is important is analytically solvable assuming $Q \gg 1$, yielding:

$$X^*_{1\ \text{important gene}} \approx \frac{-SQ(M-Q) + 2\sqrt{S(M-Q)}(Q-1+\sqrt{\gamma})}{M}. \tag{S20}$$

In particular the per-gene errors read:

$$x_0^* = \frac{\sqrt{S(M-Q)}}{\sqrt{\gamma}} \tag{S21a}$$

$$x_1^* = \sqrt{S(M-Q)} \tag{S21b}$$

$$x_2^* = \frac{-SQ(M-Q) + \sqrt{S(M-Q)}(Q-1+\sqrt{\gamma})}{M-Q}. \tag{S21c}$$

The error of the single important gene can be reduced at most by a factor of $\sqrt{\gamma}$ relative to the other co-activated genes. The x_1^* error for the other $Q-1$ genes remains the same, because we assumed that $Q \gg 1$. Interestingly, the $M-Q$ genes that need to be kept inactive suffer an increase in crosstalk as a consequence of protecting the important gene.

Every transcription factor regulates Θ genes

In the basic model we considered a regulatory scheme in which every gene has its own unique TF type. This allows for maximal flexibility in regulating each gene individually. Real gene regulatory

networks typically have fewer TFs than the number of target genes, so that at least some transcription factors regulate several genes. Here we consider a simple extension of the basic model, in which each TF regulates Θ genes (with identical binding sites) rather than one. We assume no overlap between the sets of genes regulated by various TFs, so that the total number of TFs species is now Θ times smaller than before. If Q genes should be active, then Q/Θ TF species should be present in a given condition. Assuming that $Q/\Theta \gg 1$, we can approximate $Q/\Theta - 1 \approx Q/\Theta$ as before. The only change from the basic crosstalk formulation is in x_1, because the concentration of cognate factors is now Θ times larger than before:

$$x_1^\Theta = \frac{e^{-E_a} + CS}{\frac{C}{Q/\Theta} + e^{-E_a} + CS} \tag{S22a}$$

$$x_2^\Theta = \frac{CS}{e^{-E_a} + CS}. \tag{S22b}$$

This formulation is analytically solvable, yielding

$$X_\Theta^* = \frac{Q}{M}\left(-\frac{S}{\Theta}(M-Q) + 2\sqrt{\frac{S}{\Theta}(M-Q)}\right) \tag{S23a}$$

$$x_1^{\Theta*} = \frac{\sqrt{S(M-Q)}}{\sqrt{\Theta}} \tag{S23b}$$

$$x_2^{\Theta*} = \frac{SQ}{\Theta}\left(\frac{\sqrt{\Theta}}{\sqrt{S(M-Q)}} - 1\right) \tag{S23c}$$

$$C_\Theta^* = \frac{e^{-E_a}Q(\Theta - S(M-Q))}{S^2(M-Q)Q + S(M-2Q)\Theta + \sqrt{S(M-Q)}\Theta^{3/2}}. \tag{S23d}$$

The equations for minimal crosstalk are equivalent to the basic model if we map $S \to S/\Theta$. Since crosstalk depends on $\sqrt{S}$ to first order, this amounts to crosstalk reduction by a factor of $\sqrt{\Theta}$.

For small S the leading term in the optimal concentration is

$$C_\Theta^* = \frac{1}{\sqrt{\Theta}}\frac{e^{-E_a}Q}{\sqrt{S(M-Q)}} + O(1). \tag{S24}$$

These gains in crosstalk have, however, been achieved by sacrificing the ability to regulate each gene individually: now, the smallest set of genes that can be co-activated is of size Θ. Typically, TFs might constitute $\gtrsim 10\%$ of the genes [4]; with $\Theta \sim 10$, the crosstalk could be reduced by a factor of ~ 3 at best.

Non-constant Θ

Until now, we assumed that each TF regulates exactly Θ genes. This assumption can be relaxed using numerical simulations; in particular, we considered the case where the number of genes that each TF regulates is a random variable drawn from a specified distribution. We started by defining which TF controls which sets of genes through explicit enumeration of binding site sequences. We assumed that the number of genes that a given TF regulates is approximately Poisson distributed (with mean Θ) and that all these regulated genes use the same sequence for their binding site, equal to the consensus sequence of the cognate TF. We then sample the environments in which Q out of the total of M genes are active; given the regulatory network structure, not all Q picks out of M can be realized, as is also the case with constant Θ model. The crosstalk is evaluated in

each environment exactly, by computing all thermodynamic states of all binding sites, and is subsequently averaged by Monte Carlo sampling through the possible environments. This extension to the model introduces no new parameters, so its crosstalk and regime boundaries can be straightforwardly compared to the model where Θ is constant. We find that Poisson-distributed Θ changes crosstalk at a below-percent level, and produces no notable shifts in regime boundaries, showing that our results are robust with respect to this particular distributional assumption.

Supplementary Note 2 Estimating the binding site similarity, S

Optimal packing

In real organisms, binding site sequences for different genes could depart from a random distribution (even after taking into account the statistical structure of the genomic background). For example, to achieve high specificity of regulation, we could hypothesize that binding site sequences evolved to minimize the overlap between any pair of consensus sequences. To explore the crosstalk limit under such optimal use of sequence space and contrast it with the random choice of binding sites, we synthetically constructed binding site sequences that are as distinct as possible. Specifically, our optimal codes are described by a parameter $d_{\min}$, which is the minimum required number of basepair differences between any pair of binding site sequences. This is the Hamming distance, HD, between sequences. The problem of choosing M sequences of length L such that each pair differs by at least $d_{\min}$ is not tractably solvable in general. We construct numerical approximations to these optimal codes using the following algorithm:

1. Generate all possible sequences of length L and store them in a list called $words$. Create an empty list, called $codewords$, which will store the binding site sequences.
2. Pick the first entry, s, from the list $words$, to be a binding site sequence, and append it to the list $codewords$.
3. Erase s and all of its Hamming neighbours at distance strictly less than $d_{\min}$ from the list $words$.
4. If the list $words$ is not empty, repeat from step 2. If the list $words$ is empty, stop.

When the procedure terminates, the list $codewords$ will contain binding site sequences that are separated by at least $d_{\min}$ mismatches. The outcome of this procedure depends on the initial ordering of the list of all possible sequences. The procedure is not guaranteed to generate the maximal set of sequences satisfying the Hamming distance criteria. From the list of generated binding site sequences, we obtain $P(d)$, the distribution of mismatch distances between all pairs of binding sites, and hence obtain the value of S as

$$\tilde{S}(d_{\min}) = \sum_{d \geq d_{\min}} P(d) e^{-\epsilon d}. \tag{S25}$$

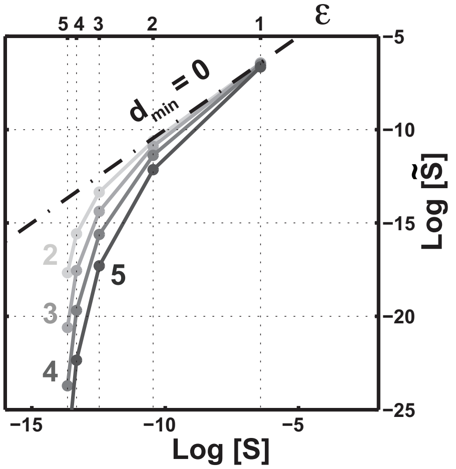

Supplementary Figure 7: **Optimal packing.** This alternative model with optimal packing of binding sites in sequence space leads to values for $\tilde{S}$ (y-axis) that can be remapped to the $S(\epsilon, L)$ (x-axis) for the random code with the mismatch energy model, $E(d) = \epsilon d$ and $L = 10$ bp binding sites (corresponding scale for ϵ shown in the top axis). Dashed lines denote equality. Optimally designed binding sites effectively decrease S. Here, their sequences are at least $d_{\min}$ bp distant from each other (gray lines = different $d_{\min}$ as indicated).

$d_{\min} = 0$ corresponds to the "random code" and results in $\tilde{S}(d_{\min} = 0) = S = (\frac{1}{4} + \frac{3}{4}e^{-\epsilon})^L$. Note that increasing $d_{\min}$ decreases the maximum possible M as sequences move further apart in sequence space whose size is fixed. A well-known upper bound on the number of sequences satisfying the Hamming distance criterion is the Singleton bound [5]: $M(d_{\min}, L) \leq 4^{L-d_{\min}+1}$. As shown in Fig. 8, with $L = 8$ and $d_{\min} = 3$, we already have $M \leq 4096$. With $L = 10$ and $d_{\min} = 4$, we have $M \leq 16384$. As L becomes smaller, the possible range of M also decreases. This suggests that prokaryotes are capable of having optimally packed binding site sequences, because they typically have $L > 10$ and $M < 10^4$. On the other hand, eukaryotes have smaller L and larger M and might not have enough sequence space to pack it optimally.

Reverse complemented sequences

We have also considered a different definition of distance between sequences that takes the double-stranded nature of DNA into account. This brings into picture the reverse complement of both sequences in question. If s_i and s_j are two sequences with reverse complements r_i and r_j respectively, this new definition of Hamming distance is

$$HD_{rc}(s_i, s_j) = \min \Big[HD(s_i, s_j), HD(r_i, s_j), HD(s_i, r_j), HD(r_i, r_j) \Big] \tag{S26}$$

where $HD(s_i, s_j)$ is the usual Hamming distance as considered previously. This restricts the sequence space much more than with the usual definition and as such, as seen in Fig. 8, we can pack fewer binding sites in the sequence space at a specific $d_{\min}$. Given that there are enough sequences under HD_{rc} measure in the sequence space, we can also ask how S changes in relative to the random code. Intuitively, S should increase since each binding site sequence also contributes its reverse complement into the pool of sequences to which TFs can bind non-cognately. Indeed,

Fig. 9, which maps S from the reverse complement code to S from a random code, shows that S

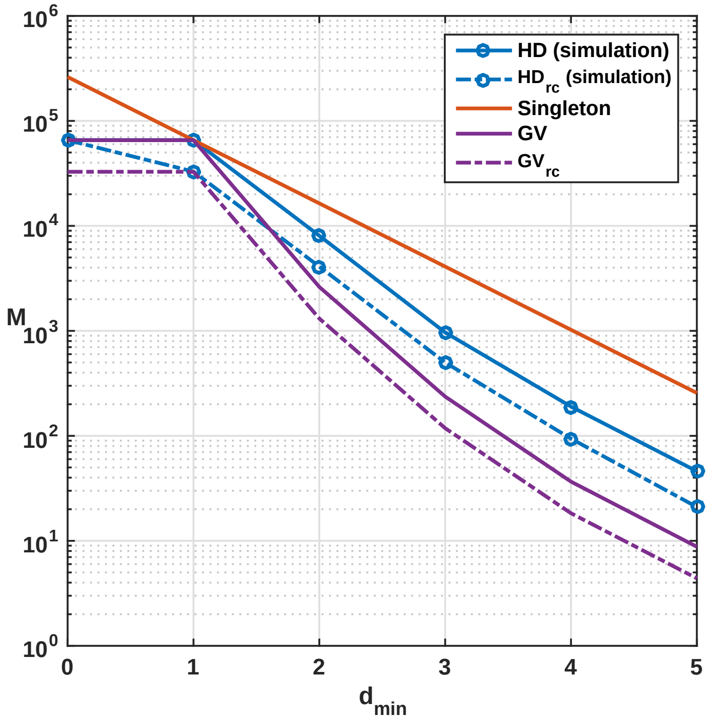

Supplementary Figure 8: **Bounds on the maximal number of binding site sequences for different $d_{\min}$ with binding sites of length $L = 8$.** Two bounds from the coding theory (Singleton upper bound and Gilbert-Varshamov (GV) lower bound [5]) are shown together with the values of M obtained by our numerical approximation procedure. These are shown both for the usual definition of distance between sequences as the Hamming distance, HD, as well as for a definition that considers the reverse complements of the sequences, HD_{rc}. For $d_{\min} = 0$ there are $M = 4^8 \approx 65000$ possible sequences where all sequence pairs are at least $d_{\min}$ distant from each other, but the number quickly decreases with increasing $d_{\min}$. From the HD to HD_{rc}, the Singleton bound doesn't change from the usual situation but the Gilbert-Varshamov (GV) bound, which takes into account the "volume of restricted ball" around each sequence, goes down. Because of stronger constraints, the number of sequences that can be packed goes down from the usual situation but only by a factor of ≈ 2.

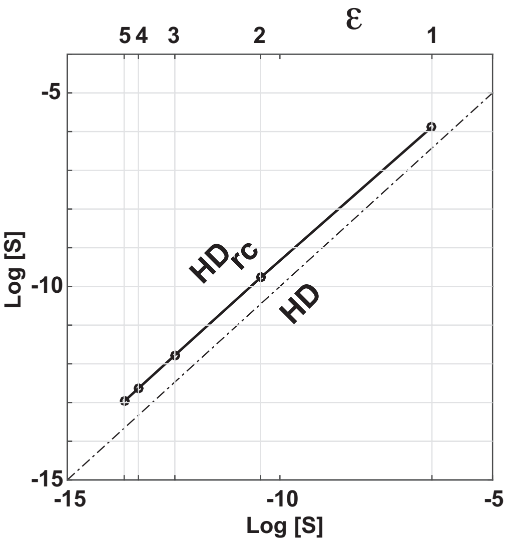

Supplementary Figure 9: **Reverse complemented sequences.** Using an alternative definition of distance (HD_{rc}) between binding site sequences, which takes into account the double-stranded nature of DNA by considering the reverse complements as well of the sequences in question, leads to values for $\tilde{S}$ (y-axis) that can be remapped to the $S(\epsilon, L)$ (x-axis) for the random code with the usual Hamming distance definition, HD. Here, we have considered $L = 8$ bp binding sites (corresponding scale for ϵ shown in the top axis). Dashed lines denote equality. This alternative definition increases S because more sequences are now found in the "shells" around the consensus to which the TF can bind on the reverse strand. S increases by about a factor of 2.

Saturating model of TF-DNA binding energy

It has been experimentally observed that the binding energy between TF and DNA saturates to some nonspecific value after a certain number of mismatches between the TF's cognate sequence and the DNA sequence in question [6]. We consider such a saturating energy model, characterized by a parameter d_0, the number of mismatches after which binding energy saturates. The binding energy is given by $E(d) = \epsilon \min(d, d_0)$. We obtain S as

$$\tilde{S}(d_0) = \sum_d P(d) e^{-E(d)}, \tag{S27}$$

where $P(d)$ is the distribution of mismatch distances between all pairs of binding sites picked at random from the sequence space. $d_0 = L$ corresponds to a mismatch model with non-saturating energy. Decreasing d_0 limits the specificity of the TF towards binding site sequences far away from the consensus and thereby increases $\tilde{S}(d_0)$.

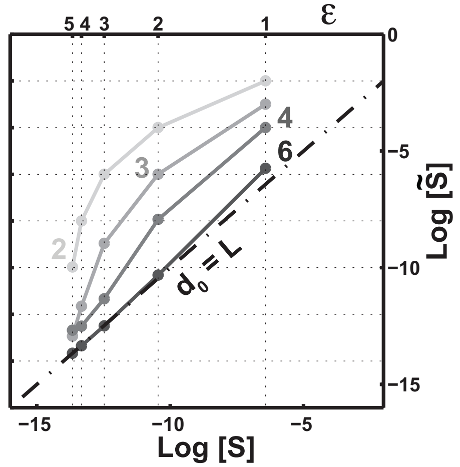

Supplementary Figure 10: **Saturating energy model.** An improved affinity model where the mismatch energy saturates after d_0 mismatches, $E(d) = \epsilon \min(d, d_0)$ (gray lines = different d_0 as indicated), effectively increases S. $d_0 \sim 4$ has been reported experimentally [6]. This alternative model leads to values for $\tilde{S}$ (y-axis) that can be remapped to the $S(\epsilon, L)$ (x-axis) for the random code with the mismatch energy model, $E(d) = \epsilon d$ and $L = 10$ bp binding sites (corresponding scale for ϵ shown in the top axis). Dashed lines denote equality.

Empirical values

We obtain organism-specific estimates of S from known databases [7, 8, 9] of the binding site sequences of different TFs. In the main text, for a particular genome, we defined S for a collection of TFs with the same mismatch penalty ϵ and binding sites of a specific constant length L. In real organisms, different TFs have different ϵ and L, making it difficult to directly calculate S for a genome. Instead we obtain a value of S for each TF by defining it as the value of S of a hypothetical genome in which all TFs have the same binding site properties (ϵ, L) as our TF. Hence, for each organism, we obtain a set of S values.

Many databases document the binding site sequences of TFs in Position Count Matrices (PCMs). The PCM of a TF with a binding site of length L is a $4 \times L$ matrix B with b_{ij} denoting the number of known TF binding site sequences that have nucleotide i in position j. One can obtain estimates of ϵ and L from B, and use them to calculate S. There are two broad ways to estimate ϵ and L (and hence, S) of a TF: (a) Information method, (b) Pseudo-count method. In (a), we calculate the information contained in the whole binding site motif and obtain an ϵ that distributes this information uniformly among all sites in an equivalent "effective" motif that has the same length as the original, but only has 0 or ϵ mismatch energy values. In (b), we obtain ϵ for all entries of the PCM and calculate an average ϵ from these entries. To handle zeros in the PCM which lead to undefined ϵ, (b) uses an arbitrary pseudo-count. Method (a) can, in contrast, avoid the use of pseudo-counts and, additionally, reproduces by construction the information content of each known motif, which is the key statistical property of TF specificity [10, 11]. Hence, we used (a) to infer S values. In both the methods, we used PCMs that have that have been constructed from at least 10 distinct binding site sequences.

Information method

In this method, we first obtain the binding site length L and also the total information I, contained in the binding site sequences of the TF.

$$I = \sum_j I_j = \sum_j \sum_i p_{ij} \log_2 \frac{p_{ij}}{q_{ij}}, \tag{S28}$$

where I_j is the information contained in position j, p_{ij} is the frequency of nucleotide i in position j, obtained in a straightforward way from B, and q_{ij} is the expected background frequency. To get rid of non-specific positions, we neglect all positions that contain information less than a certain threshold ($I_j > 0.2$ bits for position j to be considered part of the binding site). For a random genome, $q_{ij} = 0.25 \, \forall \, i, j$, resulting in

$$I = 2L + \sum_{i,j} p_{ij} \log_2 p_{ij} \tag{S29}$$

The maximum information in the motif is $2L$ bits (when $\epsilon \to \infty$) with each position contributing a maximum of 2 bits, which for finite ϵ, is reduced by an entropy term. Obtaining information per position $I_{pos} = I/L$, we infer an ϵ that uniformly distributes the information in the motif among individual positions. At a specific position j^*, without loss of generality, assume that $i = 4$ has the best binding energy ($= 0$). The probability of observing $i = 4$ at j^* is given by $p_4 = 1/Z$ while the probability of observing any of the three other possible nucleotides is given by $p_{1,2,3} = e^{-\epsilon}/Z$, with $Z = 1 + 3e^{-\epsilon}$ [12]. Hence,

$$I_{pos} = 2 + \sum_i p_i \log_2 p_i \tag{S30}$$

$$= 2 - \frac{1}{Z} \log_2 Z + 3 \frac{1}{Z \ln 2} \epsilon e^{-\epsilon} - 3 \frac{e^{-\epsilon}}{Z} \log_2 Z \tag{S31}$$

$$= 2 - \log_2 Z + 3 \frac{1}{Z \ln 2} \epsilon e^{-\epsilon} \tag{S32}$$

The mismatch energy ϵ can be obtained from the above expression, and from ϵ and L, we obtain $S(\epsilon, L) = (\frac{1}{4} + \frac{3}{4} e^{-\epsilon})^L$.

Pseudo-count method

In this method, we infer ϵ for all three non-cognate nucleotides in each position, and obtain ϵ for the TF as an average of these $3L$ values. For an arbitrary position j, as before, assume that $i = 4$ has the maximum counts ($b_{4j} > b_{ij}$, $i = 1, 2, 3$). We obtain $\epsilon_{ij} = \log \frac{b_{4j}}{b_{ij}}$ and mismatch penalty for position j as $\epsilon_j = \frac{1}{3}(\epsilon_{1j} + \epsilon_{2j} + \epsilon_{3j})$. If some entry $b_{kj} = 0$, ϵ_{kj} is undefined. To take care of this, we first add a pseudocount δ to all entries of B and obtain a modified PCM B_δ to infer ϵ. The value of δ chosen is arbitrary and it is common practice to use $\delta = 0.5$ or $\delta = 1$. As before, to get rid of non-specific positions, we consider positions that have $\epsilon_j \geq 1$. From the remaining, we take a mean to obtain $\epsilon = \frac{1}{L} \sum_j \epsilon_j$, and finally obtain $S(\epsilon, L) = (\frac{1}{4} + \frac{3}{4} e^{-\epsilon})^L$.

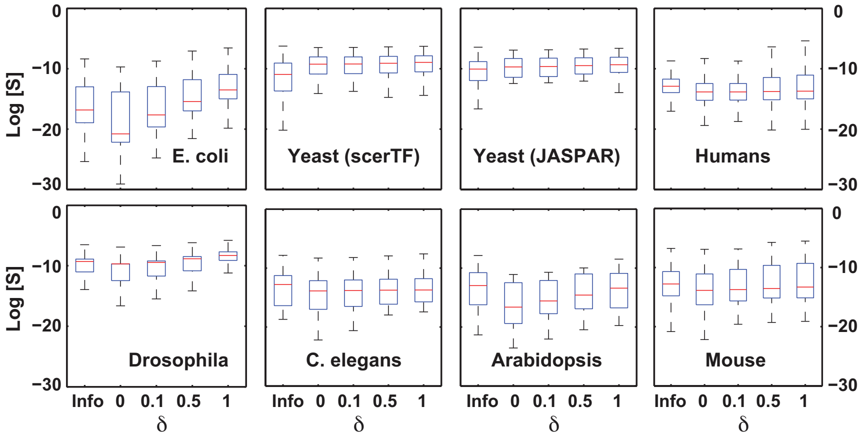

Supplementary Figure 11: **Boxplots of S for TFs from different databases.** In each panel, organism-specific (from a single database) boxplots of S are shown. The first boxplot in each panel corresponds to S values obtained from information estimates, and the remaining four correspond to S values obtained using the psuedo-count method with $\delta = 0, 0.1, 0.5, 1$ from left to right. *E. coli* TFs were obtained from RegulonDB [7] and yeast (*S. cerevisiae*) from two different databases - scerTF [9] and JASPAR [8]. All the other organism specific TFs were obtained from JASPAR. Notice that in the pseudo-count method, δ has the biggest influence on the estimates in *E. coli*. Importantly, for all other organisms, the estimates are invariant to δ and agree well with the information estimate.

Supplementary Note 3 Validity of the mean-field assumption

In computing crosstalk at given M and Q, we have made a mean-field assumption on the similarity measure S. For a given set of binding site sequences in the sequence space (total M in number), this amounts to assuming that the distribution of neighbours for each binding site comes from the same underlying distribution. For a particular selection of Q genes, for each binding site i from the M binding sites, similarity S_i can be defined using d_{ij} where $j \neq i$ indexes over the binding sites of the Q selected genes.

$$S_i = \sum_{j \neq i} e^{-\epsilon d_{ij}} \tag{S33}$$

From this, we have for crosstalk for a particular selection of Q genes,

$$\begin{aligned} X(\{S_i\}) = & \frac{1}{M}\Big[\sum_{i \in Q} x_1(S_i) + \sum_{i \in M-Q} x_2(S_i)\Big] \\ = & \frac{1}{M}\Big[\sum_{i \in Q} \frac{e^{-E_a} + CS_i}{C/Q + e^{-E_a} + CS_i} + \sum_{i \in M-Q} \frac{CS_i}{e^{-E_a} + CS_i}\Big] \end{aligned} \tag{S34}$$

where $x_1(S_i)$ and $x_2(S_i)$ depend on S_i as shown. We are interested in the mean crosstalk $X = \langle X(\{S_i\})\rangle$ over all selections of Q out of M genes, which requires us to know the full distribution of S_i. The crosstalk is then

$$X = \langle X(\{S_i\})\rangle = \frac{1}{M}\Big[\sum_{i\in Q}\langle x_1(S_i)\rangle + \sum_{i\in M-Q}\langle x_2(S_i)\rangle\Big]. \tag{S35}$$

In the mean-field assumption, we have $\langle x_1(S_i)\rangle \approx x_1(\langle S_i\rangle) = x_1(S)$ and $\langle x_2(S_i)\rangle \approx x_2(\langle S_i\rangle) = x_2(S)$, which gives us

$$X = \frac{Q}{M}x_1(S) + \frac{M-Q}{M}x_2(S). \tag{S36}$$

From this, one can obtain the optimal crosstalk X^*. To check the validity of such a mean-field assumption, we performed numerical simulations by drawing lists of M binding sites from the sequence space, computing optimal crosstalk X^*_{sim} by explicit enumeration of all thermodynamic states, and comparing this with the mean-field crosstalk X^*. In detail, we first picked M binding sites (to regulate M genes) randomly from the sequence space and held this choice fixed. Now, for each Q, we performed n_{sel} different selections of Q out of M genes. For each such selection, after computing the binding site mismatches and occupancies, we compute the crosstalk. To get the mean crosstalk for Q, we perform a Monte Carlo estimate of the mean crosstalk over these n_{sel} different selections of Q out of M genes. Figures 12 and 13 show that the mean-field crosstalk systematically over-estimates the actual crosstalk, but nevertheless remains a very good approximation to the true crosstalk.

Supplementary Note 4 Mixed models

In the baseline model we consider M genes, all of which are regulated either solely by activators or solely by repressors. Here, we consider mixed models, i.e., models that utilize repression to control one subset of genes and activation to control the other genes. Let's assume that M_A genes are regulated by activators and M_R genes are regulated by repressors, where $M = M_A + M_R$. In a particular environment, let's assume that Q genes need to be ON. Out of these, let's assume that Q_A genes are activator-regulated and Q_R genes are repressor-regulated, where $Q = Q_A + Q_R$. For activating Q genes, the number of TFs present now amounts to $T = Q_A + M_R - Q_R$: Q_A activators and $M_R - Q_R$ repressors. As before, S is the similarity of the binding sites and C the total concentration of TFs (activators+repressors). The concentration of a particular TF type, when present, will now be C/T. We assume that any non-cognate interaction ("activation out-of-context" or "repression out-of context") counts as a crosstalk error. We distinguish 4 types of per-gene crosstalk errors:

An activator-regulated gene that needs to be ON, should be bound by the cognate activator. The unbound state and any non-cognate binding (non-cognate activator or repressor) are crosstalk states:

$$x_1^A = \frac{e^{-E_a} + CS}{\frac{C}{T} + e^{-E_a} + CS} \qquad (Q_A \text{ out of } M \text{ genes}). \tag{S37}$$

An activator-regulated gene that needs to be OFF, should be unbound. Any non-cognate binding is a crosstalk state:

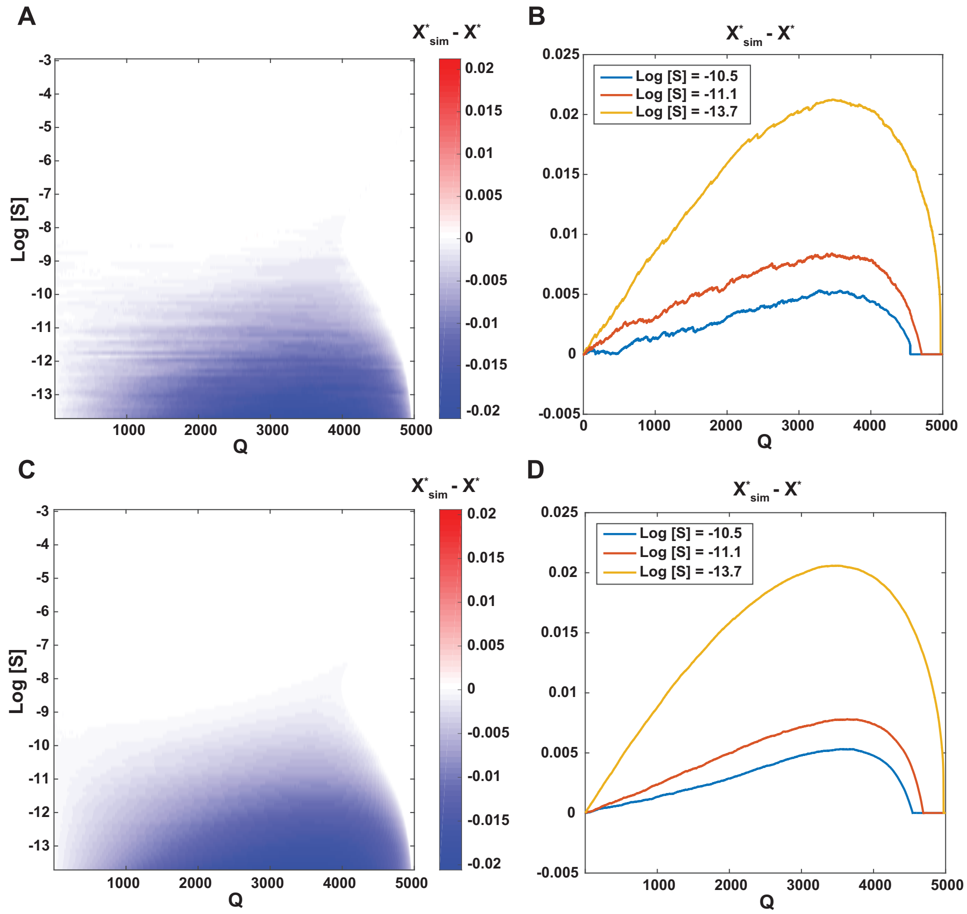

Supplementary Figure 12: **Comparison of mean-field results and numerical simulations**. On the left, we plot the difference in optimal crosstalk between simulations and the mean-field approach, $X^*_{\text{sim}} - X^*$, for different Q and S. On the right, we plot $X^*_{\text{sim}} - X^*$ against Q for three different S. Here, $M = 5000$, $L = 10$, and S has been varied by tuning ϵ. X^*_{sim} is a Monte Carlo estimate of the mean crosstalk, obtained over n_{sel} different selections of Q out of M genes. $n_{\text{sel}} = 1$ in the top row, and $n_{\text{sel}} = 30$ in the bottom row. The mean-field approach is in general a very good approximation of the simulations. The maximal crosstalk difference is less than 0.02, and decreases with increasing S.

$$x_2^A = \frac{CS}{e^{-E_a} + CS} \qquad (M_A - Q_A \text{ out of } M \text{ genes}). \tag{S38}$$

A repressor-regulated gene that needs to be ON, should be unbound. Any non-cognate binding is a crosstalk state:

$$x_1^R = \frac{CS}{e^{-E_a} + CS} \qquad (Q_R \text{ out of } M \text{ genes}). \tag{S39}$$

Lastly, a repressor-regulated gene that needs to be OFF, should be bound by the cognate repressor. The unbound state and any non-cognate binding (non-cognate repressor or activator) are crosstalk states:

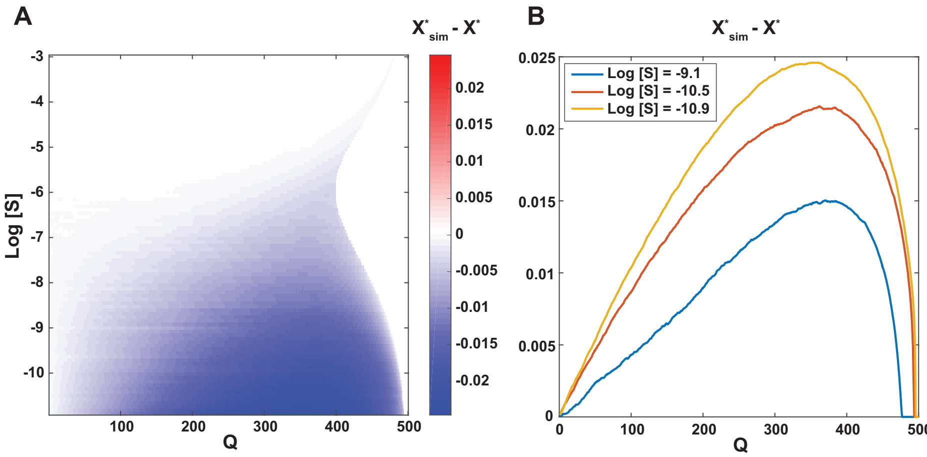

Supplementary Figure 13: **Comparison of mean-field results and numerical simulations**. On the left, we plot the difference in optimal crosstalk between simulations and the mean-field approach, $X^*_{\text{sim}} - X^*$, for different Q and S. On the right, we plot $X^*_{\text{sim}} - X^*$ against Q for three different S. Here, $M = 500$, $L = 8$, and S has been varied by tuning ϵ. X^*_{sim} is a Monte Carlo estimate of the mean crosstalk, obtained over $n_{\text{sel}} = 100$ different selections of Q out of M genes. Again, as with $M = 5000$, the mean-field approach is a very good approximation of the simulations. The maximal crosstalk difference is only slightly larger than 0.02.

$$x_2^R = \frac{e^{-E_a} + CS}{\frac{C}{T} + e^{-E_a} + CS} \qquad (M_R - Q_R \text{ out of } M \text{ genes}). \tag{S40}$$

As $x_1^A = x_2^R$ and $x_2^A = x_1^R$, the overall crosstalk error reads

$$\begin{aligned} X_{\text{mixed,full}}(Q_A, Q_R, M_A, M_R) &= x_1^A \frac{Q_A}{M} + x_2^A \frac{M_A - Q_A}{M} + x_1^R \frac{Q_R}{M} + x_2^R \frac{M_R - Q_R}{M} \\ &= x_1^A \frac{M_R + Q_A - Q_R}{M} + x_2^A \frac{M_A + Q_R - Q_A}{M} \\ &= x_1^A \frac{T}{M} + x_2^A \frac{M - T}{M} \\ &= X(Q_{\text{eff}} = T, M_{\text{eff}} = M). \end{aligned} \tag{S41}$$

Hence, given a set of (Q_A, Q_R, M_A, M_R) of the mixed model, crosstalk is same as that in an equivalent baseline activator model with $Q_{\text{eff}} = T = M_R + Q_A - Q_R$ and $M_{\text{eff}} = M = M_A + M_R$.

For a given M, different (M_A,M_R) partitions are possible, which differ in the number of genes under activator or repressor control. This can be tuned on an evolutionary timescale. Once M_A is chosen, different selections of Q genes that should be active potentially have different numbers of genes under the control of activators (Q_A) and repressors ($Q_R = Q - Q_A$). However, the optimal TF concentration C^* and the minimal crosstalk X^* only depend on the total number of TFs T.

For given M, Q, and S, we find the best possible M_A, which minimizes the crosstalk. For a particular M_A, we define the optimal crosstalk as the average optimal mixed crosstalk for all selections of Q genes out of M (averaged over different choices of Q_A),

$$X^*(M, Q, S, M_A) = \sum_{Q_A} P_{Q_A} X^*_{\text{mixed,full}}(Q_A, M, Q, S, M_A), \tag{S42}$$

where P_{Q_A} is the fraction of Q gene selections that have Q_A activated genes. We have

$$P_{Q_A} = \frac{\binom{M_A}{Q_A}\binom{M-M_A}{Q-Q_A}}{\binom{M}{Q}}, \tag{S43}$$

$$X^*_{\text{mixed}}(M,Q,S) = \min\left[X^*(M,Q,S,M_A)\right], \tag{S44}$$

$$M_A^* = \underset{M_A}{\arg\min}\, X^*(M,Q,S,M_A), \tag{S45}$$

where M_A^* is the M_A value which minimizes crosstalk for a given Q. In Fig. 14, we see that for $Q < M/2$, the best strategy is to use all activators ($M_A = M$), and for $Q \geq M/2$, the best strategy is to use all repressors; optimization of crosstalk in mixed models therefore always picks out one of the two "pure" regulatory strategies and does not yield an optimal mixed model.

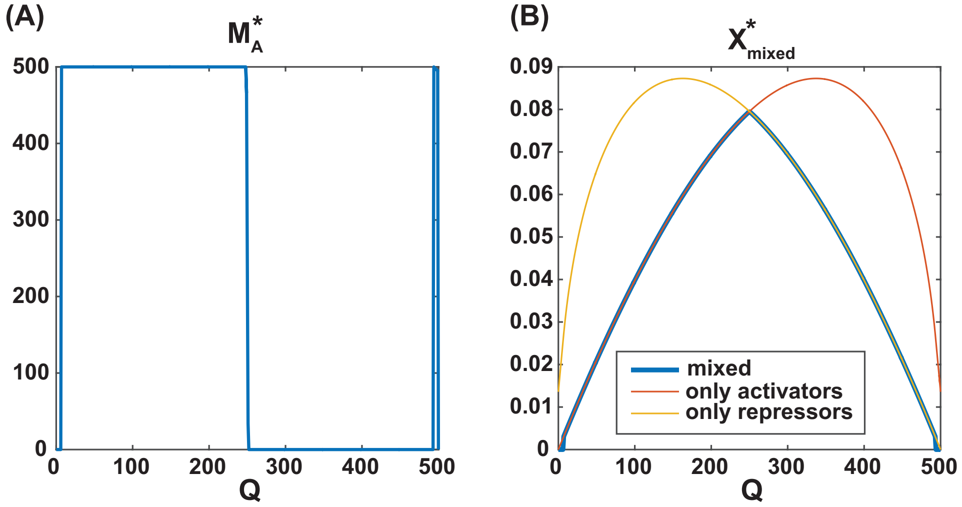

Supplementary Figure 14: **Mixed model at best M_A.** On the left, we plot the optimal number of activated genes M_A^* for different Q at $M = 500$ and $\log(S) = -10.5$. For $Q < 250$, it is best to have all genes under activator control ($M_A^* = 500$) and for $Q \geq 250$, it is best to have all genes under repressor control ($M_A^* = 0$). On the right, we plot the optimal mixed crosstalk, computed at M_A^*, and averaged over different gene selections using P_{Q_A}.

To see if the pure strategies get chosen because the activation of all genes is symmetric in all environments, we studied a simple system in which different subsets of genes are required to be activated with different probabilities. So far, when Q genes are required to be ON, each gene had the same probability, Q/M, to be among the Q out of M required genes, i.e. Q/M is the probability of each gene to be activated.

Here, we introduce two classes (1 and 2) of genes, with M_1 genes in the first class and $M_2 = M - M_1$ genes in the second class. Genes in each of the two classes have different probabilities of requiring activation across environments: P_1 for the first class and P_2 for the second class. If

$P_i > 0.5$, then genes in class i are called "hot" genes, and if $P_i < 0.5$, genes in class i are called "cold" genes. Given certain M_1, M_2, P_1, and P_2, different environments correspond to different choices of the Q genes that should be active, where Q is no longer constant as before, but a random variable with mean

$$\langle Q \rangle = P_1 M_1 + P_2 M_2.$$

In a similar fashion as before, we compute the crosstalk (at optimal C^*) for different choices of mixed models (how many class i genes are under activators or repressors). Then, we obtain the optimal (M_A,M_R) strategy among these mixed models that minimizes crosstalk. In Fig. 15, we show how this optimal strategy varies, along with $\langle Q \rangle$, as a function of P_1 and P_2 for a fixed choice of $M_1 = M_2 = 2500$. First, we note that $\langle Q \rangle$ increases in any direction that increases P_1 or P_2. In the symmetric mixed model setup, we essentially studied the system along the diagonal from $(0, 0)$ to $(1, 1)$ on the (P_1, P_2) plane (dashed white line), increasing $\langle Q \rangle$ from 0 to M. The previously studied results yielded two "pure" strategies—all activators or all repressors, depending on whether Q is bigger or smaller than $M/2$—which is consistent with the following observations in the asymmetric mixed models. When $P_1 < 0.5$ and $P_2 < 0.5$ (all genes are cold), the optimal strategy is a pure one, namely, to put all genes under activators; when $P_1 > 0.5$ and $P_2 > 0.5$ (all genes are hot), the optimal strategy is to put all genes under repressors, which is also a pure strategy. But when $P_1 > 0.5, P_2 < 0.5$ or $P_1 < 0.5, P_2 > 0.5$ (one class is hot, while the other is cold), the optimal strategy is "mixed": put hot genes under repressors and cold genes under activators. Note that not all $\langle Q \rangle$ are possible with these optimal mixed strategies. From here onwards, we study mixed models in the bottom right square of Fig. 15, where $P_1 > 0.5$ and $P_2 < 0.5$, i.e., class 1 is hot and class 2 is cold.

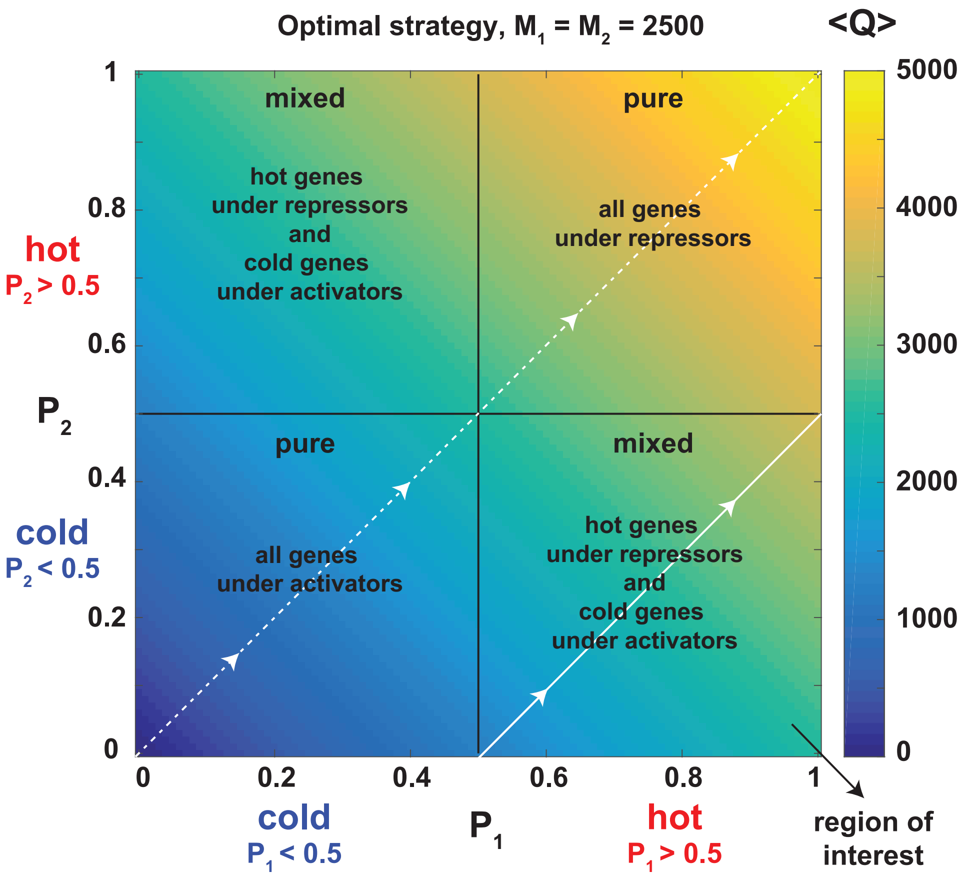

Supplementary Figure 15: **When some genes are hot and other genes are cold, the optimal mixed strategy puts hot genes under repressors and cold genes under activators.** Here we show how the optimal strategy and $\langle Q \rangle$ vary as a function of P_1 and P_2 for a fixed choice of $M_1 = M_2 = 2500$. $\langle Q \rangle$ increases in any direction that increases P_1 or P_2. When $P_1 < 0.5$ and $P_2 < 0.5$ (all genes are cold), the optimal strategy is a pure one (all genes under activator control), while when $P_1 > 0.5$ and $P_2 > 0.5$ (all genes are hot), the optimal strategy is to put all genes under repressors, which is also a pure strategy. But when $P_1 > 0.5, P_2 < 0.5$ or $P_1 < 0.5, P_2 > 0.5$ (one class is hot, while the other is cold), the optimal strategy is "mixed": hot genes are under repressor control and cold genes under activator control.

At fixed P_1 and P_2, crosstalk gains from using the optimal mixed strategy (instead of using all activators) increase with both S and the number of hot genes M_1, as shown in Fig. 16.

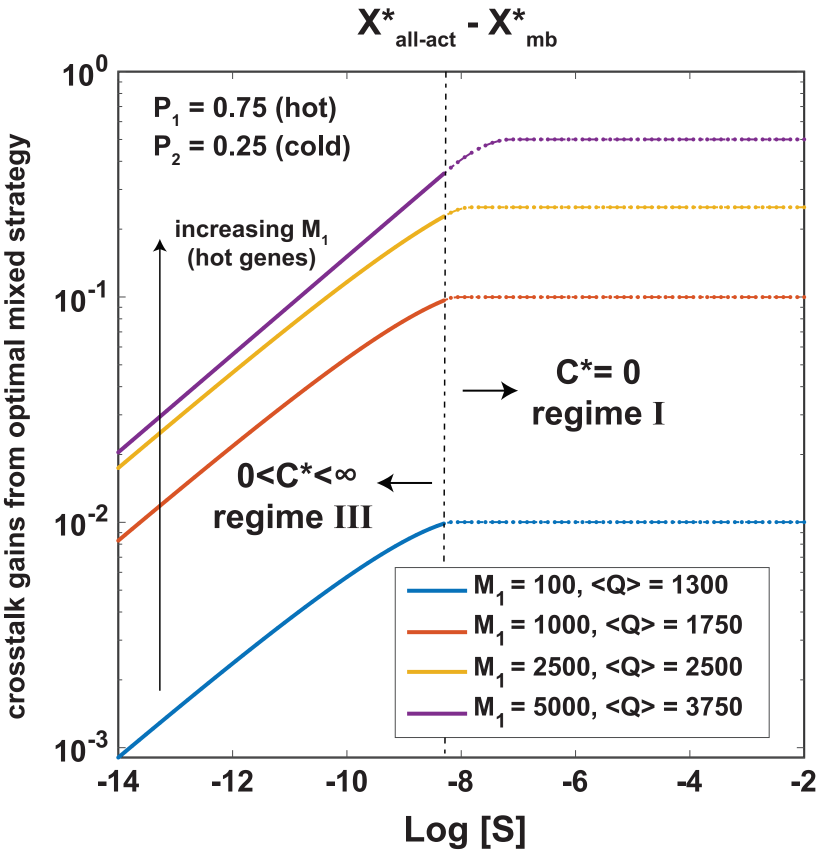

Supplementary Figure 16: **Crosstalk gains from using the optimal mixed strategy instead of all activators**. Plotted is the difference in optimal crosstalk (crosstalk gain), $X^*_{\text{all-act}} - X^*_{\text{mb}}$, between the pure strategy of using all activators and the optimal mixed strategy of putting hot genes under repressors and cold genes under activators, as a function of S, with fixed $P_1 = 0.75$ and $P_2 = 0.25$. As S increases, we cross from the regulatory regime III to regime I in which $C^* = 0$. The optimal mixed strategy becomes increasingly better (than the all activators pure strategy at reducing crosstalk) as S and M_1 increase.

In Fig. 17, we show in detail the crosstalk gains from using the optimal mixed strategy instead of the optimal pure strategy (either all activators or all repressors), for different $\langle Q\rangle$ and S, for four different $M_1 = 500, 2000, 3000$ and 4500.

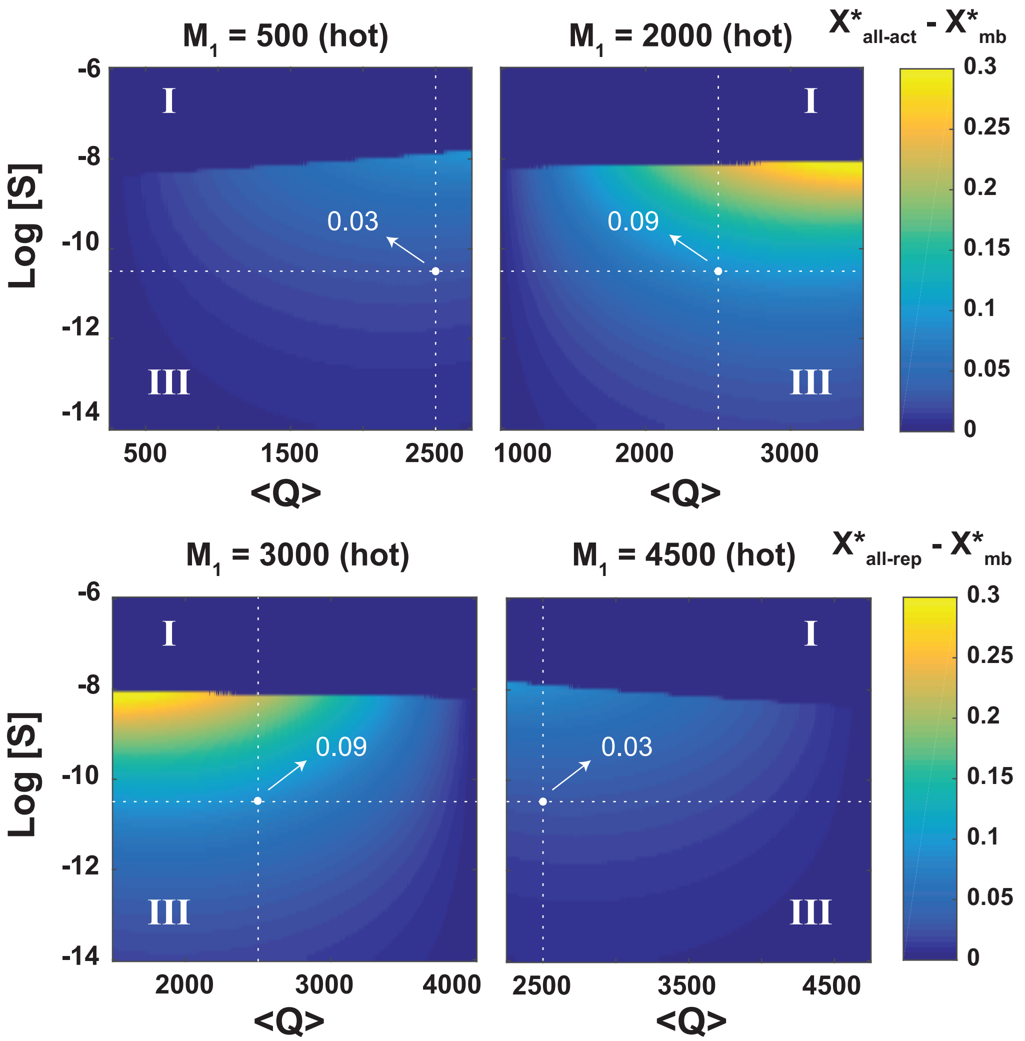

Supplementary Figure 17: **Optimal mixed strategy is increasingly better than the optimal pure strategy at intermediate M_1 and larger S, at the border of the two regimes**. Here, we plot the crosstalk gains, ($X^*_{\text{all-act}} - X^*_{\text{mb}}$ in the top row, or $X^*_{\text{all-rep}} - X^*_{\text{mb}}$ in the bottom row) from using the optimal mixed strategy instead of the optimal pure strategy as a function of the average number of genes required, $\langle Q \rangle$, and S, for different M_1. For $M_1 < M/2 = 2500$, the optimal pure strategy is to use all activators and for $M_1 > M/2 = 2500$, the optimal pure strategy is to use all repressors. Note that for $M_1 > M/2$, $X^*_{\text{all-rep}} - X^*_{\text{mb}}$ at $(\langle Q \rangle, S)$ is equal to $X^*_{\text{all-act}} - X^*_{\text{mb}}$ at $M'_1 = M - M_1 < M/2$ and $(M - \langle Q \rangle, S)$; they are laterally inverted mirror images. In general, the optimal mixed strategy gives a lower crosstalk than the optimal pure strategy for intermediate M_1. At the baseline parameters of $\langle Q \rangle = 2500, M = 5000, \log(S) = -10.5$, for $M_1 = 500$ and 4500 both, the crosstalk gain is 0.03, while for $M_1 = 2000$ and 3000, the crosstalk gain is 0.09. For a particular M_1, crosstalk gains are larger both at larger S and larger (smaller) $\langle Q \rangle$ for $M_1 > M/2$ ($M_1 < M/2$). We obtain different $\langle Q \rangle$ on the x-axes as $\langle Q \rangle = P_1 M_1 + P_2 M_2$ by varying (P_1, P_2) along the solid white line of Fig. 15 from $(0.5, 0)$ to $(1, 0.5)$.

Supplementary Note 5 Cooperative regulation

So far, we assumed a single binding site for every gene. Yet, some genes employ combinatorial regulation, with several binding sites regulated by a number of transcription factors. As a next step

in extending our model we consider cooperative regulation, where every gene has two binding sites that are bound by two copies of the same type of transcription factor.

We assume 2 binding sites per gene, with energy gap E_a between cognate-bound and unbound states. An additional energy contribution Δ is obtained if both sites are bound by cognate factors, which then interact with each other. We consider also the configuration that two noncognate factors *of the same type* bind to the double binding sites and interact with each other as well. In the limit that $\Delta \gg E_a$ once one of the sites is bound, the binding of the other becomes energetically favorable. This cooperative binding energy only applies for two molecules of the same type. Thus, if one site is bound by the cognate and the other by a noncognate molecule, cooperative interaction doesn't apply. We assume that only binding of one of the two sites induces transcription. The reasoning for this assumption is that for many bacterial and yeast genes activators are thought to work by recruiting the transcriptional machinery to the DNA [13]. Following this rationale, only one of the two sites is in the correct physical location (in bacteria, the proximal one) to do so successfully. Technically, if we assume that only one of the two sites determines transcription, for $\Delta = 0$, the cooperativity case reduces back to the basic model (Supplementary Note 1). We list the possible binding configurations of the two sites, their energies and statistical weight in Table 2.

The general case of this model, incorporating all possible binding configurations yields a 6th order equation in the TF concentration C, which we only handle numerically. The following limiting cases are however analytically solvable:

1. Limit of strong cooperativity: Assume that the cooperative interaction is strong compared to the individual protein-DNA binding energies $\Delta \gg E_a$. We can then neglect binding configurations in which only one of the sites is bound and the other is vacant, and the ones in which both are bound, but by molecules that do not interact cooperatively. That leaves us with only 3 possible binding configurations: both sites unbound, both bound by cognate TF or both bound by noncognate TF molecules of the same type with cooperative interaction (configurations 1,4 and 10 in Table 2). By proper change of variables this case can be reduced back to the basic single-binding-site model. The minimal crosstalk then reads:

$$X^*_{\text{coop}} = \frac{-Q\left(\tilde{S}(M-Q) + 2\sqrt{\tilde{S}(M-Q)}\right)}{M}, \tag{S46}$$

where $\tilde{S} = S(2\epsilon, L)$. This error is achievable with TF concentration

$$C^*_{\text{coop}} = Q\sqrt{-\frac{e^{-\Delta-2E_a}\left(\tilde{S}(M-Q)-1\right)}{\left(\tilde{S}\left(\tilde{S}Q(M-Q)+M-2Q\right)+\sqrt{\tilde{S}(M-Q)}\right)}}. \tag{S47}$$

Since the cooperative binding model allows for a binding site which is twice as long and higher total binding energy the parameters need to be correctly transformed to compare to the 1-site model. If we transform: $\tilde{S} \to S$ we obtain exactly the same minimal error as in the single-site model. By proper transformation of the energy of the unbound state $\tilde{E_a} = \Delta + 2E_a$ the TF concentration that minimizes the error is a square root of the one we had in the single-site model Eq. (S6). In similarity with the basic single-site model, here too we obtain different parameter regimes, whereas For $\tilde{S} = S(2\epsilon, L) > \frac{1}{M-Q}$ the minimal error is obtained by taking $C = 0$, namely regulation is not advantageous. While seemingly the cooperative binding is equivalent to a 1-site model which has twice as long binding site, this

	configuration	activity	crosstalk if ON	crosstalk if OFF	strong cooperativity	Energy	Weight
1	CC	ON	-		+	0	$(C/Q)^2$
2	UC	ON	-			$E_a + \Delta$	$C/Qe^{-E_a-\Delta}$
3	NC	ON	-			$\Delta + \epsilon d$	$C^2/QSe^{-\Delta}$
4	UU	OFF	+	-	+	$2E_a + \Delta$	$e^{-2E_a-\Delta}$
5	CU	OFF	+	-		$E_a + \Delta$	$C/Qe^{-E_a-\Delta}$
6	NU	OFF	+	-		$E_a + \Delta + \epsilon d$	$CSe^{-E_a-\Delta}$
7	UN	*	+	+		$E_a + \Delta + \epsilon d$	$CSe^{-E_a-\Delta}$
8	CN	*	+			$\Delta + \epsilon d$	$C^2/QSe^{-\Delta}$
9	N_xN_y	*	+	+		$\Delta + \epsilon(d_1 + d_2)$	$C^2S^2e^{-\Delta}$
10	N_xN_x	*	+	+	+	$2\epsilon d$	$\frac{C^2}{Q}S(2\epsilon, L)$

Supplementary Table 2: All possible binding configurations and the corresponding energies for a two-binding site model with cooperative interaction. 'C' denotes binding by cognate factor, 'N' - binding by noncognate and 'U' - means that the site is unbound. We distinguish between binding of noncognate molecules of the same type (N_xN_x) and different types (N_xN_y), where in the former there is also cooperative interaction between the molecules. We define the reference energetic level $E = 0$ as the state 'CC' when both sites are bound by cognate factors with cooperative interaction, such that all other energies are positive. We assume that the left binding site is the auxiliary and only the right one determines the state of activity. Note that the statistical weight of the last binding configuration N_xN_x uses $S(2\epsilon, L)$ instead of the otherwise $S(\epsilon, L)$. The column 'activity' denotes whether in the given configuration the gene is either ON, OFF or * - could be either active or inactive (possibly active in response to noncognate signal). Blank space denotes a non-existing configuration (or one which is not accounted for): these are the configurations including a cognate factor bound in the situation that it is absent because the gene should be silent. The next two columns denote whether this configuration was counted as crosstalk (+) or not (-) if the cognate transcription factor is present and the gene should be activated or if it is absent (and the gene should be silenced). The 'Strong Cooperativity' column denotes the configuration included under strong cooperativity approximation.

is not accurate. The reason is that cooperative interaction occurs only between two specific molecules, which limits the possible sequence space.

2. Limit of weak cooperativity: If $\Delta = 0$, the problem reduces to the basic single-site model.

Cooperativity with interactions between noncognate pairs

In Fig. 4 of the main text we neglected the possibility of cooperative interaction between pairs of noncognate molecules at the binding site of interest. This situation is plausible if the interaction between the molecules is facilitated by the specific binding sites. However, the molecules can also

cooperatively interact in solution before binding and then bind a noncognate site as a complex. This possibility was not taken into account in Fig. 4 (main text). In the following we repeat the calculation including this interaction too (state no. 10 in Table 2). The results are illustrated in Fig. 18. Evidently, the improvement in crosstalk owing to cooperativity is now significantly smaller.

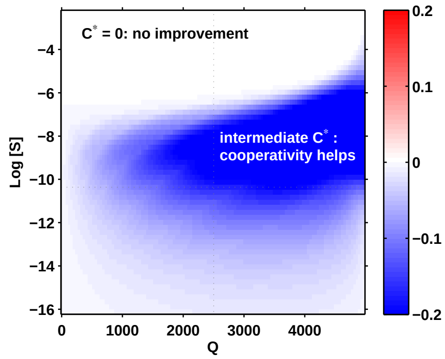

(a)

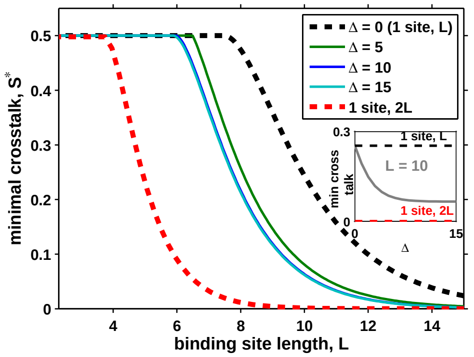

(b)

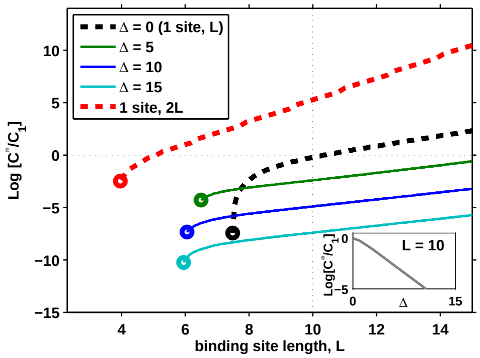

(c)

Supplementary Figure 18: **Crosstalk when any pair of the same type TFs interacts cooperatively, even if bound to noncognate site.** Here we repeat the calculation of Fig. 4 of the main text where we also account for cooperative interaction between the noncognate binders. This significantly decreases the benefit of cooperative interaction, although it still shows some improvement compared to the single-site basic model. (a): Difference in crosstalk compared to the basic model with single site, $X^*_{\text{coop}} - X^*$, where the strength of the cooperative interaction is $\Delta = 10$. One outcome of this is that the $C^* = 0$ (no regulation regime) becomes significantly larger (compare to Fig. 4B). (b): Minimal crosstalk obtained for different intensities of cooperative interaction. In contrast to the case shown in the main text Fig. 4C, where increased cooperativity always reduces crosstalk, here the improvement is limited. For example, increasing cooperativity from $\Delta = 5$ to $\Delta = 10$ brings about only a minor improvement. (c): Optimal TF concentration decreases with increased cooperativity, as in Fig. 4D. Circles denote transition to $C^* = 0$ - no regulation regime.

Supplementary Note 6 Weak global repressor

So far we only considered gene regulation by activators. Cells however also have repression mechanisms as an additional means of regulation. As a first step to account for that we incorporate in the model one type of an abundant weak global repressor that interacts with all binding sites

with sequence-independent low affinity. Non-specific repression mechanisms such as the nuclear envelope, histones and DNA methylation are thought to mitigate spurious transcription [14]. It was hypothesized that their emergence enabled the genome expansion in the transitions between prokaryotes to eukaryotes and from invertebrates to vertebrates [14]. We include an additional molecule in the model, which is found in concentration C_r and can bind all binding sites equally well with energy $0 < E_r < E_a$, namely it is more favorable than the unbound state, but not as favorable as the specific cognate activator of each site. Hence, our intuition was that such a global repressor cannot compete equally with specific binding, but it can reduce non-specific binding. The crosstalk expressions now read:

$$x_1^r = \frac{SC + C_r e^{-E_r} + e^{-E_a}}{SC + \frac{C}{Q} + C_r e^{-E_r} + e^{-E_a}} \tag{S48}$$

$$x_2^r = \frac{SC}{SC + C_r e^{-E_r} + e^{-E_a}}. \tag{S49}$$

As before, we minimize the crosstalk with respect to the TF concentration. The optimal concentration is now:

$$C_{GR}^* = -\frac{Q\left(C_r e^{-E_r} + e^{-E_a}\right)\left(\sqrt{S(M-Q)} - S(SMQ - Q(SQ+2) + M)\right)}{S\left(-M(SQ+1)^2 + SQ^2(SQ+3) + Q\right)}. \tag{S50}$$

This is the same optimal concentration C^* as in Eq. (S6) only scaled by a factor $C_r e^{-E_r} + e^{-E_a}$, instead of e^{-E_a} there. We conclude that the mere effect of a global repressor is to scale down the concentration of the specific activator. This is simply compensated for by a larger concentration of the activator. Hence, regardless of the global repressor affinity E_r and concentration C_r this additional regulatory mechanism cannot lower the crosstalk beyond what is possible with specific activators only. As before, the minimal crosstalk is:

$$X_{GR}^* = \frac{Q}{M}\left(-S(M-Q) + 2\sqrt{S(M-Q)}\right). \tag{S51}$$

Supplementary Note 7 Regulation by a combination of specific activators and specific repressors

As the global repressor examined in Supplementary Note 6 did not show any additional improvement in crosstalk, we elaborate the model further to account for specific repressors, in similarity to the specific activators. We extended the basic model (Supplementary Note 1) in which a gene had a single regulatory site and was regulated by an activator alone, to a more general model in which each gene has two regulatory sites: one compatible with a specific activator binding and the other with a specific repressor. We assume that each gene has a unique activator and unique repressor. In the basic model (Supplementary Note 1), for a gene to be silent its binding site should be vacant. The only way to achieve this was to lower the activator concentration. On the other hand, to improve activation reliability, the activator concentration, should be increased! Thus, in the simple model there seemed to be a trade-off between reliable activation and elimination of undesirable activation. The existence of a specific molecule that blocks the site from binding of other (potentially activating) molecules is thought to be a more reliable way to prevent undesired gene activation, not at the expense of the activation of other genes [15].

To be consistent with the basic model, we assume that the total concentration of all TFs (activators and repressors together) is constant C. As before, Q genes need to be activated for which Q specific activators are present. The other $M-Q$ genes need to be silent for which we now add their $M-Q$ specific repressors. All activators are found in equal concentrations $C_A/Q = \alpha * C/Q$ each. All repressors are in equal concentrations $C_R/(M-Q) = (1-\alpha) * C/(M-Q)$ each. We allow for different binding energies for the two binding sites E_a and E_r. We assume that activation can only occur by binding of an activator molecule to the 'A' site. Repression is asymmetric in the sense that binding of any molecule to the repressor site prevents binding regardless of what is bound to the activator site. Thus a gene can only be active if the repressor site is empty and the activator site is bound by an activator. See the list of all possible states of the two binding sites in Tables 3 and 4 below.

Overlapping activator and repressor binding sites

For some genes, the regulatory sites of the activator and repressor partially overlap. Another possibility is "negative cooperativity" - when one molecule repels the other. The outcome of either option is that either an activator or a repressor could be bound at any given time, but not both of them simultaneously. In Tables 3-4 all the states above the double horizontal line are such that only one site can be bound at any given time ('overlapping sites'). The additional states below the line are only possible if both sites can be bound simultaneously ('non-overlapping sites'). Fig. 19 illustrates the dependence of crosstalk on the energy E_r (energy gap between unbound and repressor-bound states) for different values of co-activated genes Q. Crosstalk is minimized for $E_r = E_a$ exactly when $Q = M - Q$, meaning equal number of activated and repressed genes. However, for other values of $Q \neq M - Q$, E_r is also not significantly different from E_a.

Supplementary Note 8 Combinatorial regulation (AND gate)

So far, we have been dealing with models in which each gene is regulated by a single type of TF, be it by a single activator, a single repressor, or multiple TFs of the same type using cooperative interactions. Here, we will consider a simple model of combinatorial regulation by a combination of two activators of different types, and compute optimal crosstalk for this setup as a function of parameters of interest.

As before, we have M genes in total, with each gene having two binding sites, corresponding to two different (cognate) TF types. For a particular gene to be ON, we need the presence of both cognate TF types, which need to occupy both binding sites. This regulatory architecture corresponds to an AND gate. We don't specify how this AND gate is implemented on the molecular level. Unlike in cooperative regulation, no additional energy gain is assumed here due to the interaction between the two TFs when bound to the DNA.

Each TF can pair with various other TFs in regulating a particular gene. In the basic activation setup, the total number of TFs, M, was equal to the total number of genes. In the combinatorial regulation setup, which is an extension of the basic activation setup, the total number of genes M will be equal to the total number of different TF-TF combinations that can exist. This will depend on the extent of combinatorial regulation, which we quantify using f, the fraction of TF-TF combinations each TF type realizes out of the theoretically maximal number of pairwise combinations it could have.

	configuration (R-site,A-site)	activity	crosstalk if ON	Energy	Weight
1	U, U	OFF	+	$E_a + E_r$	$e^{-(E_a+E_r)}$
2	U, C_A	ON	-	E_r	$\frac{C}{Q}\alpha e^{-E_r}$
3	U, N_A	*	+	$E_r + \epsilon d$	$C\alpha S e^{-E_r}$
4	U, N_R	OFF	+	$E_r + \epsilon d$	$C(1-\alpha)Se^{-E_r}$
5	C_A, U	OFF	+	$E_a + \epsilon d$	$\frac{C}{Q}\alpha S e^{-E_a}$
6	N_A, U	OFF	+	$E_a + \epsilon d$	$C\frac{Q-1}{Q}\alpha S e^{-E_a}$
7	N_R, U	OFF	+	$E_a + \epsilon d$	$C(1-\alpha)Se^{-E_a}$
8	(N_A, C_A),C_A	OFF	+	ϵd	$\frac{(C\alpha)^2}{Q}S$
9	C_A,N_A	OFF	+	$\epsilon(d_1+d_2)$	$\frac{(C\alpha)^2}{Q}S^2\frac{Q-1}{Q}$
10	N_R, C_A	OFF	+	ϵd	$\frac{C^2}{Q}S\alpha(1-\alpha)$
11	(N_A, N_R),N_A	OFF	+	$\epsilon(d_1+d_2)$	$C^2S^2\alpha\frac{Q-1}{Q}\frac{Q-\alpha}{Q}$
12	(N_R, N_A, C_A),N_R	OFF	+	$\epsilon(d_1+d_2)$	$C^2S^2(1-\alpha)$

Supplementary Table 3: All possible binding configurations, corresponding energies and statistical weights for a two-binding site (A,R)-model: a gene that needs to be activated (hence its cognate activator is present and its cognate repressor is absent). The subscripts 'A' and 'R' refer to activator and repressor. We assume that the site to which the molecule binds determines the activity state, where binding to A-site can activate the gene and binding to the R-site (even if it is an activator!) hinders activation. 'C' denotes binding by cognate factor, N - binding by noncognate and U - site is unbound. E_a and E_r are the energy gaps between unbound and cognate-bound states of the corresponding binding sites. In the upper part of the table (above the double line) we enumerate only states possible when both sites cannot be bound simultaneously (simplified model). If the two sites can be bound simultaneously, there are additional binding configurations, which are detailed below the line. The column 'crosstalk if ON' lists all binding configurations that were accounted for as crosstalk in x_1 calculation - in this case all except for no. 2 (U, C_A).

If there are T TFs in total, each TF can potentially pair with $N_{\text{int}} = f(T-1)$ other TF types, where f is the fraction of pairs each TF type realizes. This gives us $M = TN_{\text{int}}/2$, and thus $T \approx \sqrt{2M/f}$ and $N_{\text{int}} \approx \sqrt{2Mf}$. But each TF should pair with at least one other TF, so we require N_{int} ≥1. Taking both of these limits into account, we have, for N_{int}, the number of TFs each TF pairs with, and the number of total TFs T,

$$N_{\text{int}} = \max(1, \sqrt{2Mf}) \quad \text{(S52)}$$

$$T = \frac{2M}{N_{\text{int}}}. \quad \text{(S53)}$$

	configuration (R-site,A-site)	activity	crosstalk if OFF	Energy	Weight
1	U, U	OFF	-	$E_a + E_r$	$e^{-(E_a+E_r)}$
2	C_R, U	OFF	-	E_a	$\frac{C(1-\alpha)}{M-Q}e^{-E_a}$
3	N_A, U	OFF	-	$E_r + \epsilon d$	$CS\alpha e^{-E_a}$
4	N_R, U	OFF	-	$E_r + \epsilon d$	$CS(1-\alpha)e^{-E_a}$
5	U, N_A	*	+	$E_a + \epsilon d$	$CS\alpha e^{-E_r}$
6	U, (C_R, N_R)	OFF	-	$E_a + \epsilon d$	$CS(1-\alpha)e^{-E_r}$
7	C_R, (C_R N_R, N_A)	OFF	-	$E_a + \epsilon d$	$\frac{C(1-\alpha)}{M-Q}CS$
8	N_R, (C_R N_R, N_A)	OFF	-	ϵd	$C^2S^2(1-\alpha^2)$
9	N_A, (C_R N_R)	OFF	-	$\epsilon(d_1+d_2)$	$C^2S^2(1-\alpha^2)$
10	N_A, N_A	OFF	-	ϵd	$C^2S^2\alpha^2$

Supplementary Table 4: All possible binding configurations, corresponding energies and statistical weights for a two-binding site (A,R)-model: a gene that needs to be silent (hence its cognate repressor is present and its cognate activator is absent). All notation is the same as in Table 3. The column 'crosstalk if OFF' lists binding configurations that were accounted for as crosstalk in x_2 calculation - in this case only no. 5.

If each TF pairs with all other TFs, we have $f = 1$ and $N_{\text{int}} = T - 1$, which gives us $T \approx \sqrt{2M}$. We call this "perfect combinatorial regulation" because it minimizes the number of TFs needed to regulate a certain number of genes.

If each TF realizes only a fraction $1/2M < f < 1$ of its combinations, we have $N_{\text{int}} > 1$ pairs for each TF, which gives us $T \approx \sqrt{2M/f}$. We call this "imperfect combinatorial regulation".

If $f \leq 1/2M$, we have $N_{\text{int}} = 1$, which gives us $T = 2M$. We call this "worst combinatorial regulation".

As before, we will compute the optimal crosstalk when Q genes are required to be ON. Here, we compute the "typical" number of TFs present at any one time, t, by following a similar recipe as before. We have $Q = tn_{\text{int}}/2$, where n_{int} is the number of pairs per TF present at any one time. This will be smaller as there are fewer TFs present at any given time relative to the total number of TF types, i.e., $t \leq T$. As before, we have

$$n_{\text{int}} = \max(1, \sqrt{2Qf}) \tag{S54}$$

$$t = \frac{2Q}{n_{\text{int}}}. \tag{S55}$$

When $f > 1/2Q$, we have $t = \sqrt{2Q/f}$ and when $f \leq 1/2Q$, we have $t = 2Q$.

	configuration (XY)	activity	crosstalk if gene needs to be ON	crosstalk if gene needs to be OFF, C can be			Energy	Weight
			ON	X	Y	none		
1	CC	ON	-				0	$(C/t)^2$
2	UC	OFF	+		-		E_a	$e^{-E_a}(C/t)$
3	NC	ON	+		+		ϵd	$(C/t)CS$
4	CU	OFF	+	-			E_a	$e^{-E_a}(C/t)$
5	CN	ON	+	+			ϵd	$(C/t)CS$
6	UU	OFF	+	-	-	-	$2E_a$	e^{-2E_a}
7	UN	OFF	+	-	-	-	$E_a + \epsilon d$	$e^{-E_a}CS$
8	NU	OFF	+	-	-	-	$E_a + \epsilon d$	$e^{-E_a}CS$
9	N_xN_y	ON	+	+	+	+	$\epsilon(d_1 + d_2)$	$(CS)^2$
10	N_xN_x	ON	+	+	+	+	$2\epsilon d$	$(C/t)CS(2\epsilon, L)$

Supplementary Table 5: All possible binding configurations and the corresponding energies for a combinatorial regulation setup implementing an AND gate. Each gene has two binding sites which bind two different cognate TF types. The "configuration" column lists all the configurations of the two binding sites of a gene. 'C' denotes binding by cognate factor, 'N' - binding by noncognate and 'U' - means that the site is unbound. We distinguish between binding of noncognate molecules of the same type (N_xN_x) and different types (N_xN_y). The "activity" column denotes whether in the given configuration the gene is either ON or OFF. To implement the AND gate, we assume that transcription occurs (ON) only when both the binding sites are bound. The next four columns denote whether this configuration is counted as crosstalk (+) or not (-). In the leftmost column "ON", both the cognate transcription factors are present (and the gene should be ON). In the next three "OFF" columns, at least one of the cognate TFs is absent (and the gene should be OFF). In "C can be X" column, the cognate TF of only the left binding site (X) is present, in "C can be Y", the cognate TF of only the right binding site is present, and in "C can be none" column, both the cognate TFs are absent. Blank space denotes a non-existing configuration: these are the configurations including a cognate factor bound in the situation that it is absent. The column "Energy" specifies the energy of these configurations. We define the reference energetic level $E = 0$ as the state 'CC' when both sites are bound by their cognate factors, such that all other energies are positive. The column "Weight" denotes the statistical weight of the configurations, taking into account the concentrations of the relevant TFs and the energy of the configurations. Note that the statistical weight of the last binding configuration N_xN_x uses $S(2\epsilon, L)$ instead of the usual $S(\epsilon, L)$.

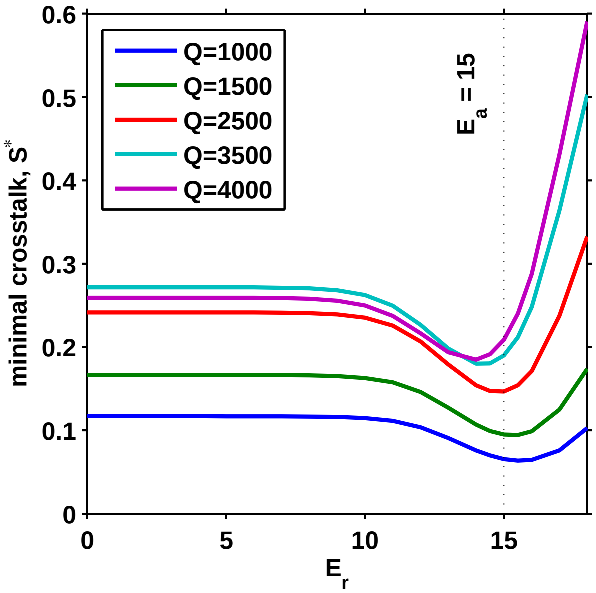

Supplementary Figure 19: **Activator-repressor overlapping binding sites, different Q values**. E_r^* - the energy gap between unbound and repressor-bound states - that minimizes crosstalk depends on the number of co-activated genes Q. Here we show numerical results for the minimal crosstalk X^* as a function of the repressor binding affinity E_r (with constant activator affinity $E_a = 15$) for different numbers of co-activated genes Q, in the model where activator and repressor binding sites overlap. We find that when the number of co-activated genes decreases (so that more genes need to be repressed) the optimal repressor affinity E_r^* increases, so that repressors more effectively bind their cognate binding sites and eliminate spurious transcription. When the number of genes that need to be activated equals the numbers of genes that need to be repressed $Q = M - Q$, we obtain that full symmetry between activator and repressor $E_r^* = E_a$ provides minimal crosstalk - this case is shown in the main text, Fig. 5. Parameters: $M = 5000$, $S = 10^{-4.5}$.

Unlike in the basic activation setup, Q genes that are required to be ON have two cognate TFs present, but genes that are required to be OFF have either none of the cognate types present, or one (but not both) of TF types present. As calculated above, we have t TFs and each TF has n_{int} combinations, while the total number of combinations it can have are N_{int}; each TF that is present therefore has $N_{\text{int}} - n_{\text{int}}$ missing combinations. The number of genes (that should be OFF) which have only one TF present can be obtained as

$$Q_1 = \frac{t(N_{\text{int}} - n_{\text{int}})}{2}. \tag{S56}$$

The number of genes with no cognate TFs present is $Q_0 = M - Q - Q_1$. In Table 5, we have listed all possible configurations for the two binding sites of a gene, along with details of crosstalk states and statistical weights. From this, we get the per-gene crosstalk for different types of genes. For genes that have both cognate TFs present (Q out of M), the per-gene crosstalk error is

$$x_{\text{both}} = 1 - \frac{(C/t)^2}{(C/t)^2 + 2e^{-E_a}(C/t) + 2(C/t)CS + 2e^{-E_a}CS + (CS)^2 + (C/t)CS(2\epsilon, L) + e^{-2E_a}}. \tag{S57}$$

For genes that have only one of the two cognate TFs present (Q_1 out of M genes), the per-gene crosstalk error is

$$x_{\text{one}} = \frac{(C/t)CS + (CS)^2 + (C/t)CS(2\epsilon, L)}{e^{-E_a}(C/t) + (C/t)CS + 2e^{-E_a}CS + (CS)^2 + (C/t)CS(2\epsilon, L) + e^{-2E_a}}. \tag{S58}$$

For genes that don't have any of their two cognate TFs present ($M - Q - Q_1$ out of M genes), the per-gene crosstalk error is

$$x_{\text{none}} = \frac{(CS)^2 + (C/t)CS(2\epsilon, L)}{2e^{-E_a}CS + (CS)^2 + (C/t)CS(2\epsilon, L) + e^{-2E_a}}. \tag{S59}$$

The total crosstalk is:

$$X = \frac{Q}{M}x_{\text{both}} + \frac{Q_1}{M}x_{\text{one}} + \left(1 - \frac{Q + Q_1}{M}\right)x_{\text{none}}. \tag{S60}$$

For a given M and f and for each (Q, S) pair, we compute the optimal concentration C^* numerically, and obtain the minimal crosstalk X^*_{comb}.

As plotted in Fig. 20, the boundaries between different regimes shift in the combinatorial setup. In particular, while at small f the "regulation regime" shrinks in the (Q, S) plane, as f increases, it expands. As f increases towards 1, the boundary between the "regulation regime" and "$C = 0$" regime moves towards larger S. In Fig. 21, we have plotted the difference in optimal crosstalk between combinatorial regulation and the basic activation setup. For $f = 0.001$, combinatorial regulation doesn't improve from the basic activation setup in terms of optimal crosstalk. But for $f = 0.01, 0.1,$ and 1, combinatorial regulation gives a lower optimal crosstalk than the basic activation setup. So, there exists a threshold in f such that for combinatorial regulation below that threshold, the "regulation regime" shrinks in comparison to the basic activation setup and performs worse. Above the threshold, the "regulation regime" expands towards larger S and gives a lower optimal crosstalk than the basic activation setup. At the baseline parameters of $Q = 2500, M = 5000$ and $\log(S) = -10.5$, optimal crosstalk for the combinatorial setups reads as $X^*_{\text{comb}} = 0.28, 0.18, 0.11$ and 0.07 for $f = 0.001, 0.01, 0.1$ and 1 respectively, compared to $X^* = 0.23$ for the basic activation setup.

This decrease in crosstalk is consistent with the reduction in the number of regulatory components (T and t, the number of TFs, see Fig. 22), as discussed in Supplementary Note 1. In the case of perfect combinatorial regulation ($f = 1$), we have roughly $\sqrt{2M}$ instead of M TF species in the basic activation setup, which is a significant reduction in the number of regulatory components. Hence, each TF now effectively controls $\Theta = M/\sqrt{2M} = \sqrt{M/2}$ genes, and so the decrease in crosstalk is expected to be roughly $\sqrt{\Theta}$ compared to the basic activation setup. For $M = 5000$ genes, this would suggest that perfect combinatorial regulation could decrease the crosstalk by ~ 7-fold over the basic model. The actual reduction in crosstalk (from 0.23 to 0.07) isn't as large because of certain differences between the combinatorial setup and Θ-genes setup of Supplementary Note 1. One major difference is that in the Θ-genes setup, the cell can only activate sets of genes of size Θ, while in the combinatorial setup, the cell has the power to activate single genes at will, albeit at

the cost of partially activating genes that aren't needed (since a considerable fraction of genes that should be OFF must have one of the two activators present) and allowing new non-cognate configurations. Fundamentally, therefore, crosstalk reduction comes from the decrease in the number of regulatory components (TF species) needed in the system, which again points to the explosion in the number of possible noncognate interactions as the crucial origin of the crosstalk. In other words, what qualitatively seems to matter is Θ, the number of regulated genes per TF, while the detailed manner in which these TFs regulate is less important for the actual numerical value of crosstalk (but *is* important for the functioning of the cell; e.g., in combinatorial regulation genes can be addressed individually, while in the model of Supplementary Note 1 they cannot be).

We also note that while near-ideal combinatorial regulation appears to be a useful strategy to reduce the crosstalk, studies of scaling laws in gene regulatory networks do not appear to be consistent with the use of such a pure combinatorial strategy. In particular, the number of TFs scales at least linearly (quadratically, in prokaryotes) with the total number of genes [4] across different organisms, while an efficient combinatorial strategy would suggest sub-linear (e.g., square-root) scaling. This clearly does not preclude the use of combinatorial regulation in some regulatory elements, but does show that even with the possible utilization of the combinatorial strategy the observed growth in the number of distinct TF species (which seems to be an important crosstalk parameter) is extensive.

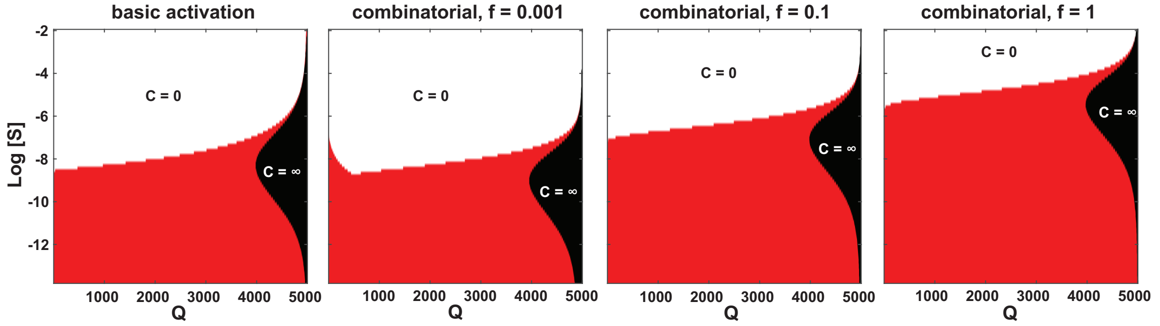

Supplementary Figure 20: **Different regimes in the (Q, S) plane for the basic and combinatorial setup.** Shifts in the regime boundaries in the basic activation setup vs. the combinatorial regulation setup. In the leftmost panel, we show the regimes for the basic activation setup. In the other panels, we show the regimes for the combinatorial setup for $f = 0.001, 0.1$, and 1, respectively, from left to right. For $f = 0.001$, the "regulation regime" is slightly smaller than in the basic activation setup. As f increases, the "regulation regime" increases in size (and is bigger than in the basic activation setup) and the boundary with $C = 0$ is pushed higher towards larger S.

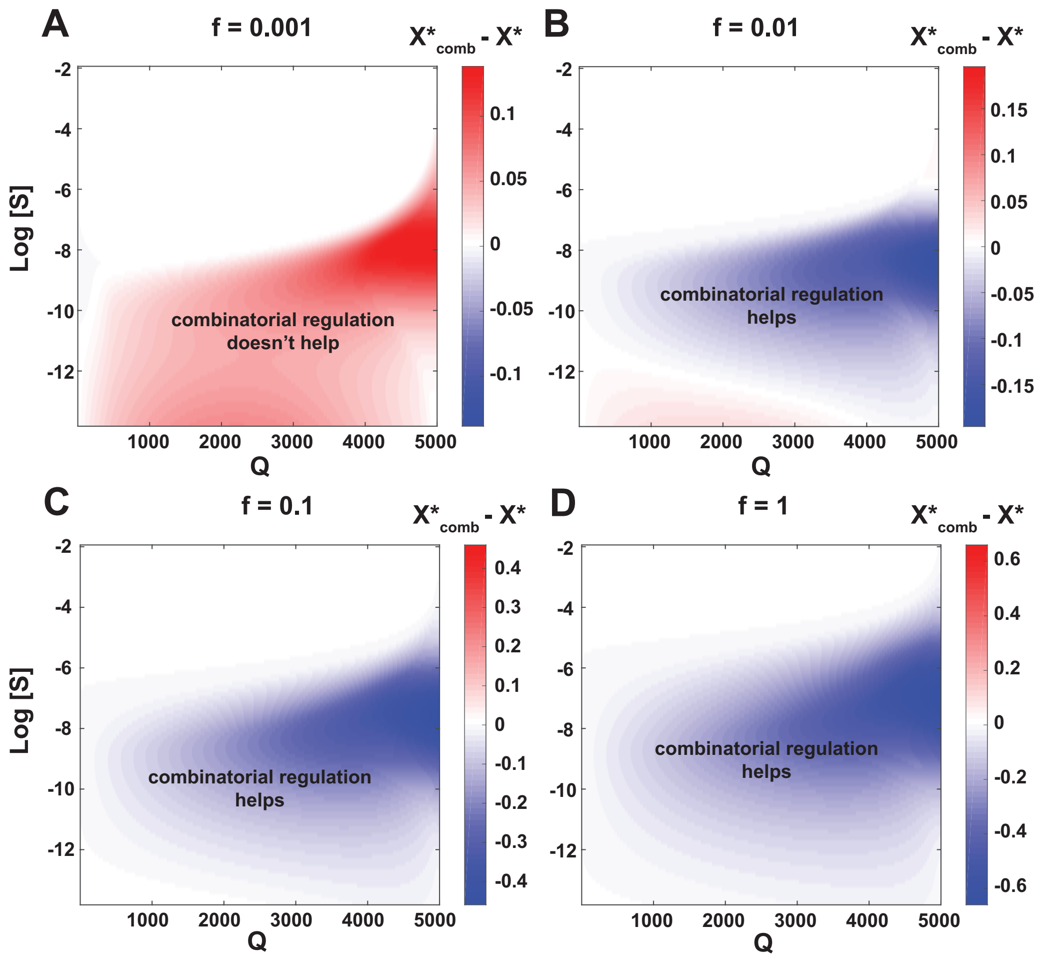

Supplementary Figure 21: **Difference in minimal crosstalk between combinatorial setup and the basic activation setup for different f.** Panel (a) shows $f = 0.001$, where combinatorial regulation underperforms the basic regulation setup. (b,c,d) Increasing values of f ($f = 0.01, 0.1, 1$, respectively) can lower the crosstalk relative to the basic setup. At baseline parameters ($Q = 2500, M = 5000$ and $\log(S) = -10.5$), minimal crosstalk for the combinatorial setups reads $X^*_{\text{comb}} = 0.28, 0.18, 0.11$ and 0.07 for $f = 0.001, 0.01, 0.1$ and 1 respectively, compared to $X^* = 0.23$ for the basic activation setup.

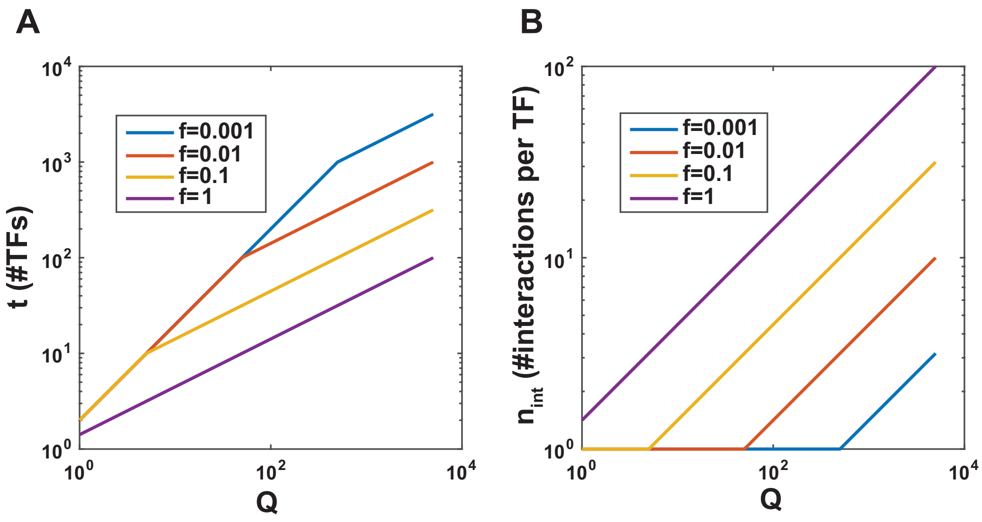

Supplementary Figure 22: **Scaling of the typical number of TFs present (t) and number of interactions per TF (n_{int}) as a function of Q for different f.** For each f, for Q smaller than some threshold value which depends on f, the number of TFs t varies as $Q = 2t$ and the number of interactions per TF n is constant at 1. For all Q greater than this threshold value, $\log n$ increases linearly with $\log Q$ (n changes with Q in a power-law fashion).

Supplementary Note 9 Alternative crosstalk definition

In the basic setup presented in the main text, we considered "activation out-of-context"—i.e., activation by the binding of a noncognate TF when the cognate TF is present (but not bound)—to be a crosstalk state. Our reasoning was motivated by viewing transcriptional regulation as a signal transmission apparatus. In this interpretation, gene activation by a noncognate TF amounts to generating a response (transcriptional activity) to a wrong input signal. Consequently, this should count as crosstalk, despite the fact that (by chance) the correct signal was simultaneously present in the cell. This is perhaps easiest to appreciate if one considers more realistic setups in which genes are not simply "ON" and "OFF", but can be quantitatively regulated by the level of their cognate TF. In such a model, there might be two TFs present and varying in concentration as a function of time: one cognate for the gene of interest and one not. In this case it is clear that the correct response of the gene is to track the changes in the cognate TF, and not to simply be expressed in a constant "ON" state; consequently, tracking the noncognate TF due to crosstalk is obviously an error, even if the cognate TF is present at the same time.

One could, however, argue that "activation-out-of-context" shouldn't be considered as an error state. If the presence or absence of TF signals is a binary variable and if the binary response is defined solely by the state of transcriptional activity (activation/inactivation of gene), then when the presence of the signal matches the response state, the regulation outcome is correct, irrespective of the molecular details on the promoter. For example, for a gene whose cognate TF is present, activation by any means (either by cognate or noncognate binding) is the correct response. In this scenario, the "out-of-context activation" is actually what one might call beneficial crosstalk: here, noncognate TF can be seen as helping to activate the gene when the cognate TF is also present. For a gene whose cognate TF is absent, activation is still an incorrect response, like before.

Hence, $x_2(i)$ retains the same expression, but $x_1(i)$ changes to

$$x_1(i) = \frac{e^{-E_a}}{C_i + e^{-E_a} + \sum_{j \neq i} C_j e^{-\epsilon d_{ij}}}. \tag{S61}$$

As shown in Fig. 23, optimizing C results in three distinct regulatory regimes, like in the default basic setup. For small S in the regulation regime, the optimal C is given to the leading order by:

$$C^* \quad \sim \quad \frac{e^{-E_a}}{\sqrt{S}} \frac{Q}{\sqrt{M-Q}} \tag{S62}$$

The minimal crosstalk error at the optimal concentration C^* is given by

$$X^* = -SQ + 2\frac{Q}{M}\sqrt{S(M-Q)(1+SQ)} \tag{S63}$$

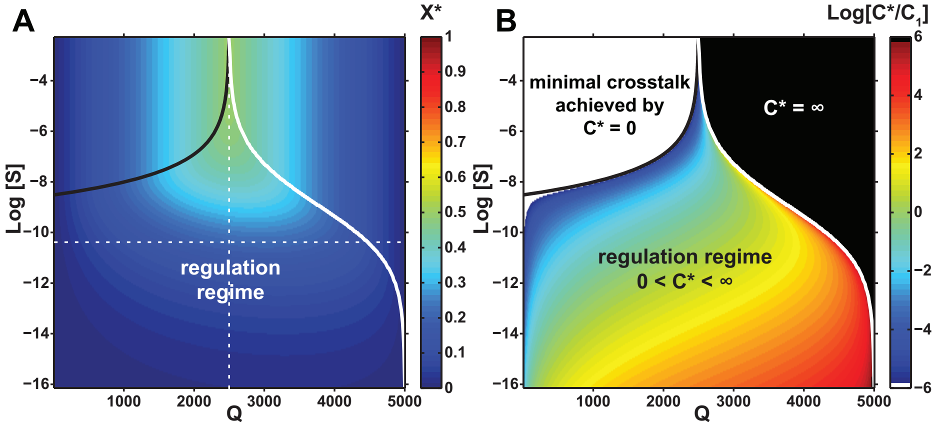

Supplementary Figure 23: **Basic model with alternative crosstalk definition also exhibits three distinct regulation regimes.** The alternative definition does not count "activation out-of-context" as an error state. **(a)** Minimal crosstalk error, X^*, shown in color, as a function of the number of coactivated genes Q, and binding site similarity S. **(b)** Optimal TF concentration C^*, that minimizes the crosstalk, relative to C_0, the optimal concentration at the baseline parameters (see main text).

Supplementary References

[1] P. H. Von Hippel and O. G. Berg. On the specificity of DNA-protein interactions. *Proceedings of the National Academy of Sciences*, 83(6):1608, 1986.

[2] Ulrich Gerland, J. David Moroz, and Terence Hwa. Physical constraints and functional characteristics of transcription factor DNA interaction. *Proceedings of the National Academy of Sciences*, 99(19):12015–12020, 2002.

[3] Franz M. Weinert, Robert C. Brewster, Mattias Rydenfelt, Rob Phillips, and Willem K. Kegel. Scaling of gene expression with transcription-factor fugacity. *Physical Review Letters*, 113(25):258101, December 2014.

[4] Erik van Nimwegen. Scaling laws in the functional content of genomes. *Trends in Genetics*, 19(9):479–484, September 2003.

[5] *Error Control Coding*. Prentice Hall, Upper Saddle River, N.J, 2 edition edition, June 2004.

[6] Sebastian J. Maerkl and Stephen R. Quake. A Systems approach to measuring the binding energy landscapes of transcription factors. *Science*, 315(5809):233–237, January 2007.

[7] S. Gama-Castro, H. Salgado, M. Peralta-Gil, A. Santos-Zavaleta, L. Muiz-Rascado, H. Solano-Lira, V. Jimenez-Jacinto, V. Weiss, J. S. Garca-Sotelo, A. Lpez-Fuentes, et al. RegulonDB version 7.0: transcriptional regulation of Escherichia coli K-12 integrated within genetic sensory response units (Gensor Units). *Nucleic Acids Research*, 39(suppl 1):D98–D105, 2011.

[8] Anthony Mathelier, Xiaobei Zhao, Allen W. Zhang, François Parcy, Rebecca Worsley-Hunt, David J. Arenillas, Sorana Buchman, Chih-yu Chen, Alice Chou, Hans Ienasescu, Jonathan Lim, Casper Shyr, Ge Tan, Michelle Zhou, Boris Lenhard, Albin Sandelin, and Wyeth W. Wasserman. JASPAR 2014: an extensively expanded and updated open-access database of transcription factor binding profiles. *Nucleic Acids Research*, page gkt997, November 2013.

[9] Aaron T. Spivak and Gary D. Stormo. ScerTF: a comprehensive database of benchmarked position weight matrices for Saccharomyces species. *Nucleic Acids Research*, 40(D1):D162–D168, January 2012.

[10] Zeba Wunderlich and Leonid A. Mirny. Different gene regulation strategies revealed by analysis of binding motifs. *Trends in Genetics*, 25(10):434–440, October 2009.

[11] Thomas D. Schneider, Gary D. Stormo, Larry Gold, and Andrzej Ehrenfeucht. Information content of binding sites on nucleotide sequences. *Journal of Molecular Biology*, 188(3):415–431, April 1986.

[12] Otto G. Berg and Peter H. von Hippel. Selection of DNA binding sites by regulatory proteins: Statistical-mechanical theory and application to operators and promoters. *Journal of Molecular Biology*, 193(4):723–743, February 1987.

[13] Mark Ptashne and Alexander Gann. Transcriptional activation by recruitment. *Nature*, 386(6625):569–577, 1997.

[14] Adrian P. Bird. Gene number, noise reduction and biological complexity. *Trends in Genetics*, 11(3):94–100, March 1995.

[15] Guy Shinar, Erez Dekel, Tsvi Tlusty, and Uri Alon. Rules for biological regulation based on error minimization. *Proceedings of the National Academy of Sciences*, 103(11):3999–4004, March 2006.
